# Co-activator binding protein PIMT mediates TNF-α induced insulin resistance in skeletal muscle via the transcriptional down-regulation of MEF2A and GLUT4

**DOI:** 10.1038/srep15197

**Published:** 2015-10-15

**Authors:** Vasundhara Kain, Bandish Kapadia, Navin Viswakarma, Sriram Seshadri, Bhumika Prajapati, Prasant K Jena, Chandana Lakshmi Teja Meda, Maitreyi Subramanian, Sashidhara Kaimal Suraj, Sireesh T Kumar, Phanithi Prakash Babu, Bayar Thimmapaya, Janardan K Reddy, Kishore V. L. Parsa, Parimal Misra

**Affiliations:** 1 Department of Biology, Dr. Reddy’s Institute of Life Sciences, University of Hyderabad Campus, Hyderabad, Telangana, India; 2 Institute of Science, Nirma University, Sarkhej-Gandhinagar Highway, Ahmedabad, Gujarat, India; 3 Department of Biotechnology, School of Life Sciences, University of Hyderabad, Hyderabad, Telangana, India; 4 Department of Microbiology and Immunology, Feinberg School of Medicine, Northwestern University, Chicago, Illinois, United States of America; 5 Department of Pathology, Feinberg School of Medicine, Northwestern University, Chicago, Illinois, United States of America

## Abstract

The mechanisms underlying inflammation induced insulin resistance are poorly understood. Here, we report that the expression of PIMT, a transcriptional co-activator binding protein, was up-regulated in the soleus muscle of high sucrose diet (HSD) induced insulin resistant rats and TNF-α exposed cultured myoblasts. Moreover, TNF-α induced phosphorylation of PIMT at the ERK1/2 target site Ser^298^. Wild type (WT) PIMT or phospho-mimic Ser298Asp mutant but not phospho-deficient Ser298Ala PIMT mutant abrogated insulin stimulated glucose uptake by L6 myotubes and neonatal rat skeletal myoblasts. Whereas, PIMT knock down relieved TNF-α inhibited insulin signaling. Mechanistic analysis revealed that PIMT differentially regulated the expression of GLUT4, MEF2A, PGC-1α and HDAC5 in cultured cells and skeletal muscle of Wistar rats. Further characterization showed that PIMT was recruited to GLUT4, MEF2A and HDAC5 promoters and overexpression of PIMT abolished the activity of WT but not MEF2A binding defective mutant GLUT4 promoter. Collectively, we conclude that PIMT mediates TNF-α induced insulin resistance at the skeletal muscle via the transcriptional modulation of GLUT4, MEF2A, PGC-1α and HDAC5 genes.

The incidence of Type 2 diabetes (T2D) is steadily increasing and it may progress into an epidemic if not controlled in time^1^,^2^,^3^. Energy-rich diets containing high levels of fat and refined carbohydrates such as sucrose and fructose along with sedentary lifestyles are believed to be the most critical factors contributing to this pandemic^4^,^5^,^6^,^7^. Insulin resistance, a hallmark of T2D, is characterized by the impaired action of insulin at peripheral tissues such as adipose and skeletal muscle^8^,^9^,^10^,^11^,^12^,^13^. Data over the last two decades provide undeniable evidence that insulin resistance is of inflammatory origin^11^,^14^,^15^,^16^,^17^,^18^. It is well documented that the expression of pro-inflammatory cytokines like TNF-α is locally enhanced in skeletal muscle and adipose tissues of humans and animals with insulin resistance and/or diabetes^17^,^18^,^19^,^20^,^21^,^22^,^23^,^24^. Many independent researchers using cell based studies and different animals models established that TNF-α induces the activity of MAPKs (ERK1/2, p38 and JNK) and other kinases such as IKKβ, PKC, mTOR and its downstream effector, S6K which in turn phoshorylate IRS1 at Ser^307^ resulting in interruption of insulin signaling^25^,^26^,^27^,^28^,^29^,^30^,^31^,^32^. In addition, TNF-α was also shown to influence transcription profile majorly through NFКB activation^33^. For instance, TNF-α signaling through its receptor TNFR1 suppressed insulin sensitivity by the inactivation of the key energy sensor AMPK via the transcriptional upregulation of PP2C^34^. Acute treatment of 3T3L1 adipocytes with TNF-α led to the degradation of IRS1 through IL-6/SOCS3 axis^35^,^36^,^37^. Importantly, prolonged exposure of human adipose cells to TNF-α was shown to reduce both transcript and protein levels of GLUT4 and IRS, key molecules in insulin mediated glucose homeostasis^33^,^38^,^39^. As noted above, chronic elevation of pro-inflammatory cytokines (TNF-α) is implicated in insulin resistance, however the underlying mechanisms in general and specifically the molecular basis of TNF-α mediated GLUT4 repression are unclear. Restoration of insulin sensitivity is a major challenge in the treatment of diabetes. The withdrawal of the potent insulin sensitizer, Avandia due to its side effects demands the discovery of a new insulin sensitizer with minimal side effects^40^.

PIMT/NCoA6IP (**P**RIP **I**nteracting protein with **M**ethyl **T**ransferase domain), a transcriptional co-activator (PRIP/NCoA6) interacting protein (NCoA6IP), is expressed ubiquitously including liver, kidney and skeletal muscle tissues^41^. PIMT is a RNA methlytransferase: it hypermethylates small nuclear RNA (snRNA), small nucleolar RNA (snoRNA) and selenoprotein mRNAs^42^,^43^,^44^,^45^,^46^. Further, PIMT regulates transcriptional apparatus via direct interaction with HAT (Histone Acetyl Transferase)-containing transcriptional co-activators CBP and p300 and non-HAT containing co-activators PBP/Med1 and PRIP^47^. PIMT bridges HAT and non-HAT containing transcriptional co-activators and facilitates transcription suggesting that PIMT is a component of the nuclear receptor signaling cascade and may have a general role in the control of chromatin structure and modulation of transcription, a role that is evident by the embryonic lethality of PIMT knockout mice^48^ .

Recently, we have shown that 1) PIMT deficiency in the liver impaired hepatic gluconeogenesis 2) ERK2-mediated phosphorylation of PIMT at Ser^298^ is essential for hepatic gluconeogenesis 3) Hyperthyroidism induces PIMT Ser^298^ phosphorylation and enhances PEPCK expression resulting hyperglycemia in rats^49^. These findings pointed towards an important role for PIMT/phospho PIMT in glucose homeostasis prompting us to systematically characterize the functional implications of PIMT in skeletal muscle tissue, a key metabolic tissue involved in glucose metabolism.

In the current study, we observed that the expression of PIMT was up-regulated in the soleus muscle of high sucrose diet (HSD) induced insulin resistant rats and TNF-α treated cultured skeletal muscle cells. PIMT overexpression abrogated insulin stimulated glucose uptake by L6 myotubes and neonatal rat skeletal myoblasts. In contrast, knockdown of PIMT in L6 myoblasts enhanced insulin sensitivity and relieved TNF-α induced inhibition of glucose uptake. Data obtained from cultured skeletal muscle cells and adenoviral infected rat skeletal muscle tissue demonstrate that PIMT inhibited insulin stimulated glucose uptake *via* the transcriptional modulation of several genes associated with skeletal muscle glucose uptake, particularly GLUT4, MEF2A, PGC-1α and HDAC5. Importantly, TNF-α induced phosphorylation of PIMT at Ser^298^ was essential for PIMT-mediated suppression of GLUT4 expression and glucose uptake. Taken together, we show that PIMT/phospho PIMT facilitates TNF-α induced insulin resistance in skeletal muscle.

## Results

### TNF-α induced PIMT abrogates insulin-stimulated glucose uptake in skeletal muscle cells

**Figure 1:**
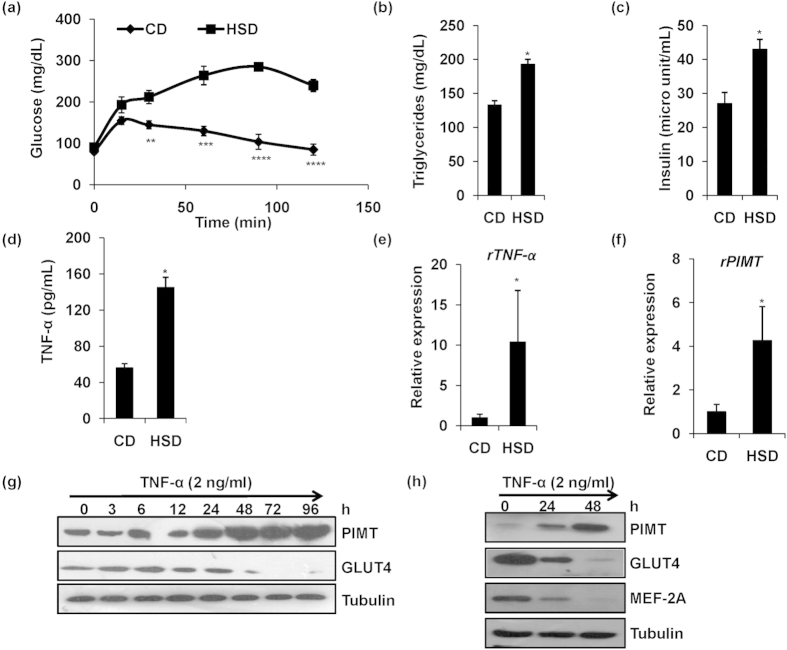
HSD–induced TNF-α augments PIMT expression in skeletal muscle. (**a**) Blood glucose levels during OGTT (2gkg^−1^) in control diet (CD) and HSD (high sucrose diet) fed rats (n = 6). Values are shown as mean ± SD; **p < 0.01, ***p < 0.005 and ****p < 0.001 for CD vs HSD group using Student’s *t-*test. (**b**–**d**) Serum levels of triglycerides (**b**), insulin (**c**) and TNF-α (**d**) of rats fed with CD and HSD (n = 6). Values are shown as mean ± SD.; *p < 0.05 versus CD using student’s *t* test. (**e**,**f**) mRNA expression of rattus TNF-α (**e**) and rattus PIMT (**f**) in soleus muscle of CD and HSD fed rats (n = 3). Values are shown as mean ± SD.; *p < 0.05 versus CD using student’s *t* test. (**g**,**h**) Western blotting for PIMT and GLUT4 in L6 myoblasts (**g**) and PIMT, GLUT4 and MEF2A in neonatal skeletal myoblasts (**h**) treated with TNF-α (2 ng/ml) for the indicated time points. The cropped blots were run under the same experimental conditions. The full-length blots are included in Supplemental Fig. 8.

**Figure 2:**
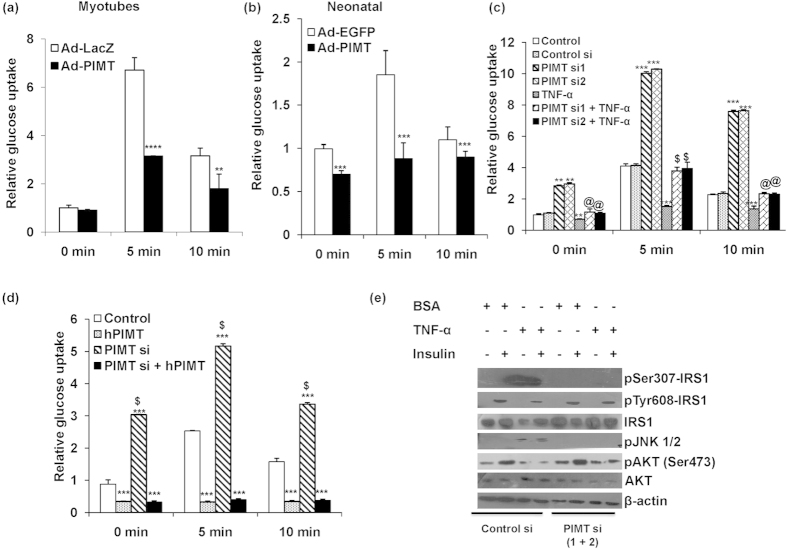
PIMT abrogates insulin stimulated glucose uptake by skeletal muscle cells. (**a**,**b**) Mean basal and insulin stimulated (5 min and 10 min) uptake of 2-NBDG by L6, L6 myotubes (**a**) and neonatal skeletal myoblasts (**b**) infected with Ad-PIMT. Values are shown as mean ± SD after normalizing with the corresponding protein content and expressed relative to basal of control cells which was set to 1; **p < 0.01, ***p < 0.005, ****p < 0.001 versus corresponding control cells (two way ANOVA). (**c**) Basal and insulin stimulated (5 and 10 min) uptake of 2-NBDG by L6 myoblasts treated with TNF-α and/or PIMT siRNA. Values are shown as mean ± SD after normalizing with the corresponding protein content and expressed relative to basal of control cells which was set to 1; **p < 0.01, ***p < 0.005 versus corresponding control cells (two way ANOVA); ^@^*p* < 0.01, ^$^*p* < 0.005 versus corresponding TNF-α treated cells (two way ANOVA). (**d**) Basal and insulin stimulated (5 and 10 min) uptake of 2-NBDG by L6 myoblasts transfected with PIMT siRNA and/or pcDNA3.1 PIMT (hPIMT). Values are shown as mean ± SD after normalizing with the corresponding protein content and expressed relative to basal control which was set to 1; ***p < 0.005 versus control cells; ^$^*p* < 0.001 versus corresponding PIMT transfected cells (two way ANOVA). (**e**) Western blotting to detect levels of phosho-IRS1^S307^, phospho-IRS1^Y608^, total IRS1, pJNK1/2, pAkt, Total Akt and β-actin in siRNA (control or PIMT) tranfected L6 myoblasts cultured with BSA or TNF-α (48 h), treated with or without insulin (100nM) for 30 min. The cropped blots were run under the same experimental conditions. The full-length blots are included in Supplemental Fig. 8.

In our previous study utilizing liver specific PIMT knockout mice, we have demonstrated that PIMT augments hepatic gluconeogenesis, an important aspect of glucose homeostasis^49^. Thus, to further explore the functional role of PIMT in glucose metabolism, we studied the involvement of PIMT in glucose homeostatic mechanisms in skeletal muscle. It is well documented that the expression of TNF-α is enhanced in the skeletal muscle and cultured myocytes from insulin resistant/diabetic humans and genetically or diet induced insulin resistant animals^17^,^19^,^24^,^50^. Further, the involvement of TNF-α in inducing insulin resistance at adipose and skeletal muscle tissues is widely recognized^19^,^20^,^24^ (Supplemental Fig. 1). Thus, we used high sucrose diet (HSD) induced insulin resistant rats and TNF-α induced L6 cells (skeletal muscle cell line) as the model systems to study the involvement of PIMT in skeletal muscle insulin resistance. HSD fed rats expectedly displayed impaired glucose tolerance (Fig. 1a) with hypertriglyceridemia (Fig. 1b) and hyperinsulinemia (Fig. 1c). Moreover serum (Fig. 1d) and local skeletal muscle TNF-α levels (Fig. 1e) were elevated. We also observed that the expression of PIMT was up-regulated in the soleus muscle of HSD fed rats (Fig. 1f). We have followed up these observations in cultured L6 cells and found that PIMT was readily detectable in L6 myoblasts and proteins levels were found to be elevated 24 h post TNF-α treatment (Fig. 1g). Likewise, PIMT protein was also enhanced in TNF-α exposed primary rat neonatal skeletal myoblasts (Fig. 1h). In parallel, we observed a decline in the protein levels of GLUT4 and MEF2A, the key transcription factor of GLUT4 (Fig. 1g,h). Elevated levels of PIMT in skeletal muscle of HSD fed rats and TNF-α treated myoblasts suggested a potential role for PIMT in the skeletal muscle insulin resistance. To test this, we transfected L6 myoblasts with empty vector or PIMT-Flag construct and 48 h later we measured insulin stimulated glucose uptake using fluorescent 2-NBDG dye. Forced expression of PIMT in L6 myoblasts abolished uptake of 2-NBDG both at 5 and 10 min post insulin stimulation (Supplemental Fig. 2a). Likewise, infection of L6 myotubes with Ad-PIMT also suppressed insulin stimulated glucose uptake (Fig. 2a). Similar results were also obtained in primary rat neonatal skeletal myoblasts (Fig. 2b). To confirm these findings, we next performed loss of function experiments. Here, we transfected L6 cells with two different siRNAs targeting PIMT and examined their impact on insulin stimulated glucose uptake. Knockdown of PIMT enhanced basal glucose uptake by ~3 fold while ablation of PIMT augmented insulin stimulated glucose uptake both at 5 and 10 min post stimulation (Fig. 2c, Supplemental Fig. 2b). Importantly, ablation of PIMT relieved TNF-α mediated inhibition of insulin-stimulated glucose uptake (Fig. 2c). However, glucose uptake was restored to the level of control but not PIMT siRNA transfected cells. This may be due to TNF-α mediated but PIMT-independent inhibitory mechanisms. To rule out the possibility that the enhanced glucose uptake noticed in PIMT knockdown cells was due to any non-specific effects of PIMT siRNA, we restored PIMT expression by co-transfecting rat origin L6 myoblasts with the siRNA-resistant human PIMT construct. The enhanced glucose uptake in PIMT siRNA transfected cells was completely abolished in siRNA and PIMT cDNA co-transfected cells (Fig. 2d). To assess the functional role of PIMT in insulin sensitivity, we investigated the effect of PIMT expression on insulin signaling. Consistent with literature^25^,^26^,^39^, IRS1^Ser307^ and JNK1/2 phosphorylation were significantly enhanced upon TNF-α treatment in control siRNA transfected L6 myoblasts (Fig. 2e). Interestingly, transient knock down of PIMT in L6 myoblasts robustly inhibited TNF-α induced IRS1^Ser307^ and JNK1/2 phosphorylation. Further, the phosphorylation levels of IRS1^Tyr608^ and Akt^Ser473^ were also noted to be restored at least partially in TNF-α treated PIMT ablated L6 myoblasts. In contrast, infection of L6 myoblasts with Ad-PIMT enhanced TNF-α mediated inhibition of insulin stimulated IRS1^Tyr608^ and Akt^Ser473^ phosphorylation (Supplemental Fig. 2c). Further, overexpression of PIMT in L6 myoblasts enhanced IRS1^Ser307^ and p38 phosphorylation (Supplemental Fig. 2C). Collectively, the above findings demonstrate that PIMT mediates TNF-α-induced insulin resistance in skeletal muscle cells.

### TNF-α induced phosphorylation of PIMT at Ser^298^ is required for the inhibition of insulin stimulated glucose uptake

**Figure 3:**
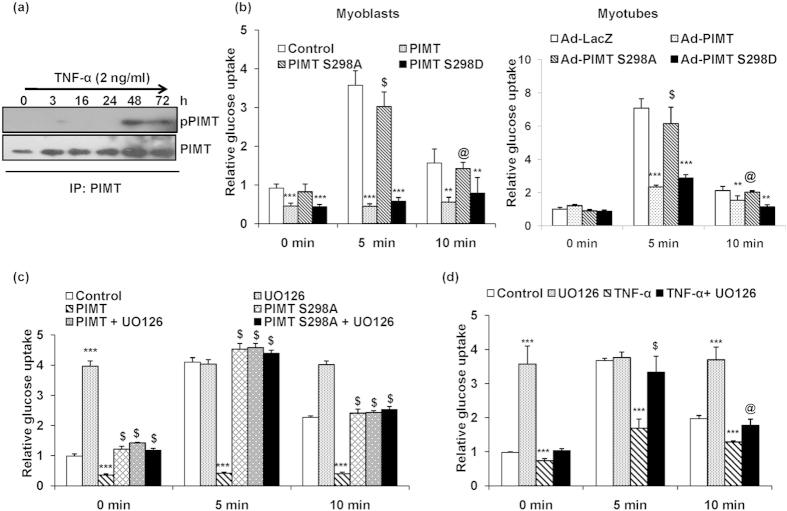
TNF-α induced ERK mediated phosphorylation of PIMT is required for PIMT dependent inhibition of glucose uptake. (**a**) Western blotting to detect phospho-PIMT (using anti-MPM2) and total PIMT treated in TNF-α treated L6 myoblasts. The cropped blots were run under the same experimental conditions. The full-length blots are included in Supplemental Fig. 8. (**b**) Basal and insulin stimulated (5 and 10 min) uptake of 2-NBDG by PIMT or mutant PIMT transfected L6 myoblasts (left panel) or L6 myotubes infected with Ad-PIMT EGFP or Ad-PIMT mutants (right panel). Values are shown as mean ± SD after normalizing with the corresponding protein content and expressed relative to corresponding control cells which was set to 1; **p < 0.01, ***p < 0.005 versus corresponding control cells, ^@^*p* < 0.01, ^$^*p* < 0.005 versus corresponding PIMT over-expressing cells (two way ANOVA). (**c**) Basal and insulin stimulated (5 and 10 min) uptake of 2-NBDG by L6 myoblasts transfected with PIMT or its mutants treated with UO126 where indicated. Values are shown as mean ± SD after normalizing with the corresponding protein content and expressed relative to basal of control cells which was set to 1; ***p < 0.005, versus corresponding control cells, ^$^*p* < 0.005 versus corresponding PIMT transfected cells (two way ANOVA). (**d**) Basal and insulin stimulated (5 and 10 min) uptake of 2-NBDG by L6 myoblasts treated with TNF-α or UO126 or both. Values are shown as mean ± SD after normalizing with the corresponding protein content and expressed relative to basal of controls cells which was set to 1; ***p < 0.005, versus corresponding control cells, ^@^*p* < 0.01,^$^*p* < 0.005 versus corresponding TNF-α treated cells (two way ANOVA).

It is well known that TNF-α induces the activation of ERK1/2 in multiple cell types including myoblasts and the activation of ERK1/2 pathway is directly linked to skeletal muscle insulin resistance^51^,^52^. We have recently shown that ERK2 phosphorylates PIMT at Ser^298^ position which was essential for PIMT mediated increase in the expression of the rate limiting gluconeogenic enzyme, PEPCK and thus hepatic glucose output^49^. Thus, we next examined the effect of TNF-α on the phosphorylation status of PIMT using ERK1/2 substrate antibody, MPM2. Using PIMT Ser298Ala mutant, we have shown that the ERK1/2-dependent MPM2 signals of PIMT are specific to Ser^298^ 49. No detectable TNF-α-induced phosphorylation of PIMT was observed at earlier time points however robust phosphorylation of PIMT was detected 48 h post stimulation which sustained up to 72 h post TNF-α treatment (Fig. 3a). We next assessed the functional consequence of TNF-α-induced phosphorylation of PIMT. To do this, we overexpressed wild type (WT) PIMT or phospho-deficient (Ser298Ala) or phospho-mimic (Ser298Asp) mutants of PIMT in L6 myoblasts and examined their effect on insulin-stimulated glucose uptake. Overexpression of WT PIMT abolished insulin stimulated glucose uptake (Fig. 3b, left panel). However, the phospho-deficient Ser298Ala mutant failed to alter insulin stimulated glucose uptake. Similar data were obtained in rat primary neonatal skeletal myoblasts (Supplemental Fig. 3). On the other hand, consistent with our previous data^49^, the phospho-mimic mutant of PIMT (Ser298Asp), similar to WT PIMT, inhibited insulin stimulated uptake of glucose by L6 myoblasts (Fig. 3b). Parallel experiments showed that the differential effects of the WT and mutants of PIMT on glucose uptake were not due to differences in their expression levels (data not shown). Further, infection of differentiated L6 myotubes with Ad-PIMT or Ad-PIMT Ser298Asp but not Ad-PIMT Ser298Ala suppressed insulin stimulated glucose uptake (Fig. 3b, right panel). The expression of WT and PIMT mutants in L6 myotubes was comparable (Supplemental Fig. 4). Moreover, we observed that the pharmacological blockade of MEK/ERK1/2 pathway by U0126 alleviated both PIMT and TNF-α mediated inhibition of insulin stimulated glucose uptake (Fig. 3c,d). Taken together, data indicate that TNF-α induced phosphorylation of PIMT at Ser^298^ is required for inhibition of insulin stimulated glucose uptake.

### PIMT abolishes insulin-stimulated glucose uptake via the differential regulation of MEF2A, GLUT4 and HDAC5 expression

**Figure 4:**
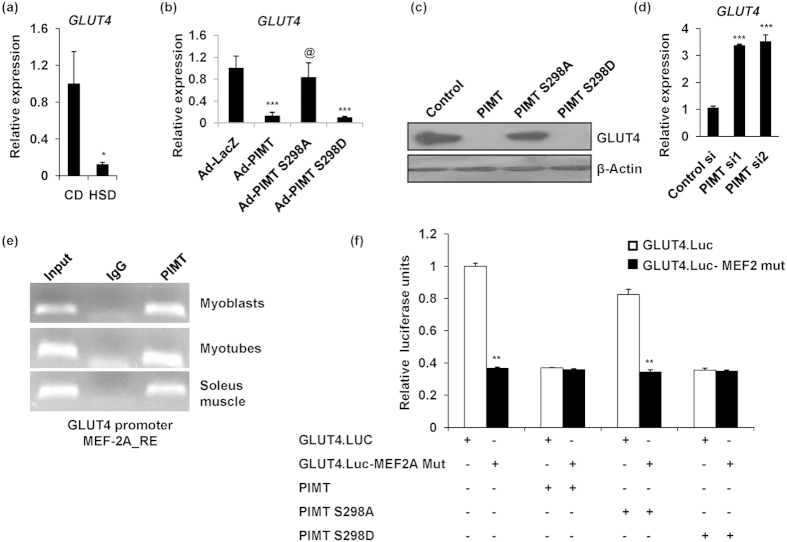
PIMT attenuates GLUT4 expression in Ser^298^ phosphorylation dependent manner. (**a**) mRNA expression of rattus GLUT4 in soleus muscle of CD and HSD fed rats (n = 3). Values are shown as mean ± SD. *p < 0.05 versus CD using Student’s *t-*test. (**b**) mRNA expression of rattus GLUT4 in L6 myotubes infected with PIMT (wt and Ser^298^ mutants). Values are shown as mean ± SD; ***p < 0.005 versus Ad-LacZ infected cells, ^@^*p* < 0.01 versus Ad-PIMT infected cells (two way ANOVA). (**c**) Western blotting for GLUT4 in wild type and Ser^298^ mutant PIMT transfected L6 myoblasts. The cropped blots were run under the same experimental conditions. The full-length blots are included in Supplemental Fig. 8. (**d**) mRNA expression of rattus GLUT4 in L6 myoblasts transfected with PIMT siRNA. Values are shown as the mean ± SD; ***p < 0.005 versus control cells (two way ANOVA). (**e**) Chromatin immunoprecipitation was performed in lysates of L6 myoblasts, L6myotubes and soleus muscle of Wistar rats using Anti-PIMT or mock Anti-goat IgG on the MEF2A response element (MEF2A_RE) of GLUT4 promoter. The full-length gel pictures are included in Supplemental Fig. 8. (**f**) Wild-type (GLUT4.Luc.) and MEF2 binding defective mutant (GLUT4.Luc.MEF2 mut) GLUT4 promoter luciferase activity in HEK293T cells transfected with WT or PIMT mutants. Values represent mean ± SD and are expressed relative to the vector transfected cells; **p <  0.01 versus vector transfected cells (two way ANOVA).

**Figure 5:**
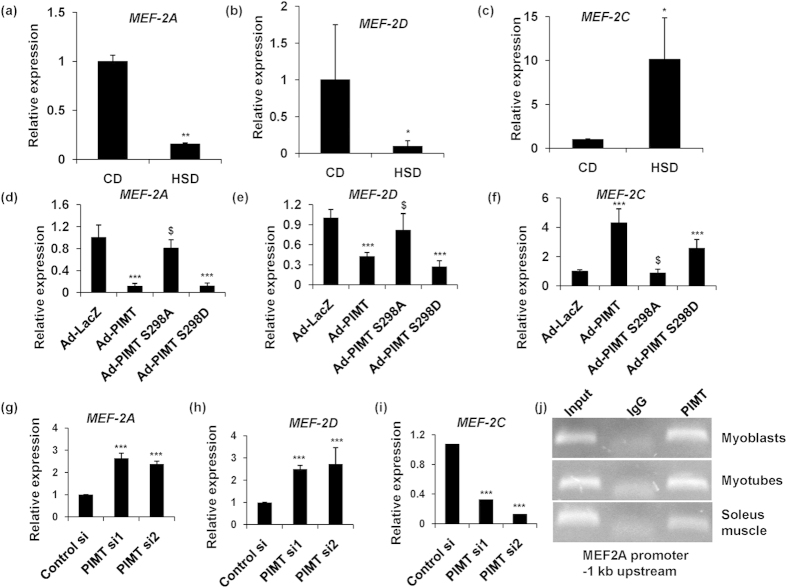
Overexpression of PIMT inhibits MEF2A expression in Ser^298^ phosphorylation dependent manner. (**a**–**c**) mRNA expression of rattus MEF2A (**a**), MEF2D (**b**) and MEF2C (**c**) in soleus muscle of CD and HSD fed rats (n = 3). Values are shown as mean ± SD; *p < 0.05, **p < 0.01 versus CD using Student’s *t-*test. (d-f) mRNA expression of rattus MEF2A (**d**), MEF2D (**e**) and MEF2C (**f**) in L6 myotubes infected with Ad-PIMT and Ad-PIMT Ser^298^ mutants. Values are shown as the mean ± SD; ***p < 0.005 versus LacZ infected L6 myotubes, ^$^*p* < 0.05 versus Ad-PIMT infected myotubes (two way ANOVA). (**g**–**i**) mRNA expression of rattus MEF2A (**g**), MEF2D (**h**) and MEF2C (**i**) in L6 cells transfected with PIMT siRNA. Values are shown as the mean ± SD; ***p < 0.005 versus control L6 myoblasts (two way ANOVA). (**j**) Chromatin immunoprecipitation was performed in lysates of L6 myoblasts, L6 myotubes and soleus muscle of Wistar rats using Anti-PIMT or mock Anti-goat IgG of −1 kb upstream region of MEF2A promoter. The full-length gel pictures are included in Supplemental Fig. 8.

**Figure 6:**
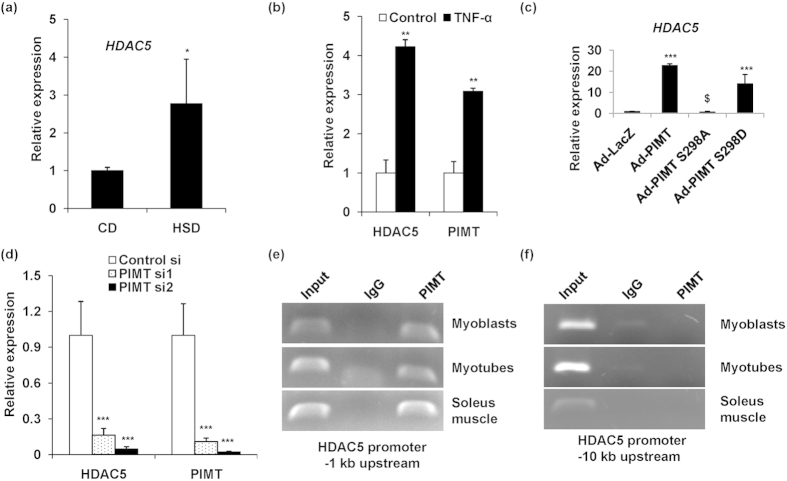
TNF-α induced PIMT up-regulates HDAC5 expression in cultured skeletal muscle cells. (**a**) mRNA expression of rattus HDAC5 in soleus muscle of CD and HSD fed rats (n = 3). Values are shown as mean ± SD. *p < 0.05 versus CD using Student’s *t* test. (**b**) mRNA expression of rattus HDAC5 and PIMT in TNF-α treated L6 myoblasts. Values are shown as mean ± SD; **p < 0.01 versus control treated using Student’s *t* test. (**c**) mRNA expression of rattus HDAC5 in L6 myotubes infected with Ad-PIMT or Ad-PIMT Ser^298^ mutants. Values are shown as the mean ± SD; ***p < 0.005 versus Ad-LacZ infected L6 myotubes, ^$^*p* < 0.05 versus PIMT (WT) infected myotubes (two way ANOVA). (**d**) mRNA expression of rattus HDAC5 and PIMT transfected with PIMT siRNA in L6 myoblasts. Values are shown as mean ± SD; ***p < 0.005 versus control L6 myoblasts (two way ANOVA). (**e**,**f**) Chromatin immunoprecipitation was performed in lysates of L6 myoblasts, L6 myotubes and soleus muscle using Anti-PIMT or mock Anti-goat IgG of HDAC5 promoter (−1 kb (**e**) and −10 kb (**f**) upstream). The full-length gel pictures are included in Supplemental Fig. 8.

Having observed that PIMT abrogates insulin-stimulated glucose uptake, we next investigated the underlying mechanisms. Consistent with the literature^23^,^53^,^54^, we observed that GLUT4 expression was down-regulated in TNF-α exposed L6 myoblasts, neonatal rat skeletal myoblasts and soleus muscle of HSD fed rats (Figs 1g,h and 4a). Moreover, PIMT overexpression caused a robust inhibition of insulin-stimulated glucose uptake by L6 cells suggesting that a key player such as GLUT4 may be the likely downstream target of PIMT. To test this, we overexpressed WT PIMT or Ser^298^ mutants of PIMT in L6 myotubes by adenoviral infection and examined the expression of GLUT4 by qPCR. Forced expression of WT PIMT or phospho-mimic but not phospho-deficient mutant of PIMT caused a dramatic inhibition of GLUT4 transcript (Fig. 4b). Similar results were obtained in L6 myoblasts as well (Supplemental Fig. 5a). Further, ectopic expression of PIMT or phospho-mimic mutant but not phospho-deficient mutant robustly reduced GLUT4 protein levels (Fig. 4c and Supplemental Fig. 5b). In contrast, knockdown of PIMT by two different individual siRNAs resulted in ~3.2 fold up-regulation of GLUT4 mRNA levels (Fig. 4d). Further investigation showed that PIMT was recruited to rat GLUT4 promoter in L6 myoblasts/myotubes and soleus muscle of Wistar rats (Fig. 4e) and overexpression of WT PIMT or PIMT Ser298Asp but not Ser298Ala mutant inhibited GLUT4 promoter activity (Fig. 4f). GLUT4 transcription is controlled through the co-operative function of two distinct regulatory elements, domain 1 and MEF2 domain and each element is required for GLUT4 transcription^55^. The MEF2 domain binds to transcription factors MEF2A and MEF2D, of which MEF2A seems to be more critical for human GLUT4 promoter activity^56^. Treatment of primary neonatal skeletal myoblasts with TNF-α resulted in reduced MEF2A protein levels (Fig. 1h). Thus, we wondered whether PIMT represses GLUT4 transcription via the inhibition of MEF2A expression. To examine this, *first*, we studied the expression of different members of MEF2 family by qPCR analysis. Feeding rats with HSD caused dramatic reduction in the levels of MEF2A and MEF2D (Fig. 5a,b) whereas the expression of another isoform, MEF2C was robustly enhanced (Fig. 5c) in the soleus muscle of HSD fed rats. Importantly, infection with Ad-PIMT or Ad-PIMT Ser298Asp but not PIMT Ser298Ala resulted in a dramatic reduction of MEF2A and MEF2D expression in L6 myotubes (Fig. 5d,e). In contrast, MEF2C expression was pronouncedly increased in Ad-PIMT or Ad-PIMT Ser298Asp mutant infected cells (Fig. 5f). Similar results were obtained PIMT over-expressed L6 myoblasts (Supplemental Fig. 6a–c). In contrast, knockdown of PIMT augmented MEF2A and MEF2D but down-regulated MEF2C mRNA levels (Fig. 5g–i) in L6 myoblasts. Moreover, PIMT was found to be recruited to −1 kb upstream region of MEF2A promoter in L6 myoblasts/myotubes and soleus muscle of Wistar rats (Fig. 5j). *Second*, we performed promoter-reporter assays with WT and mutant GLUT4 promoter which is defective in binding to MEF2A. Mutant promoter construct consistently showed reduced promoter activity compared to WT GLUT4 promoter (Fig. 4f). Overexpression of WT and phospho-mimic mutant of PIMT but not phospho-deficient mutant of PIMT inhibited the activity of WT GLUT4 promoter. In contrast, WT or PIMT mutants failed to alter the activity of GLUT4 promoter containing defective MEF2 binding site (Fig. 4f). MEF2A, the pivotal player in GLUT4 transcription is reported to interact with transcriptional co-repressor HDAC5^57^,^58^,^59^. Following AMPK-dependent phosphorylation of HDAC5, MEF2A-HDAC5 association is lost allowing chromatin remodeling and MEF2A mediated transcription to proceed^60^. Thus, we next investigated the effect of PIMT on HDAC5 expression. While the expression of HDAC5 was modestly enhanced in the soleus muscle of HSD rats and TNF-α challenged L6 myoblasts (Fig. 6a,b), ablation of PIMT by siRNA markedly down-regulated HDAC5 expression (Fig. 6d) in L6 myoblasts. In contrast, infection of L6 myotubes with Ad-PIMT or Ad-PIMT Ser298Asp but not Ad-PIMT Ser298Ala led to a striking increase in HDAC5 expression (Fig. 6c). Further, PIMT was recruited to −1 kb upstream (Fig. 6e) but not −10 kb upstream region (Fig. 6f) of HDAC5 promoter in L6 myoblasts/myotubes and soleus muscle of Wistar rats. Taken together, the above data indicate that PIMT represses GLUT4 expression via the modulation of the expression of MEF2 isoforms and HDAC5.

### PIMT dependent inhibition of insulin-stimulated glucose uptake involves Med1 and PGC-1α

**Figure 7:**
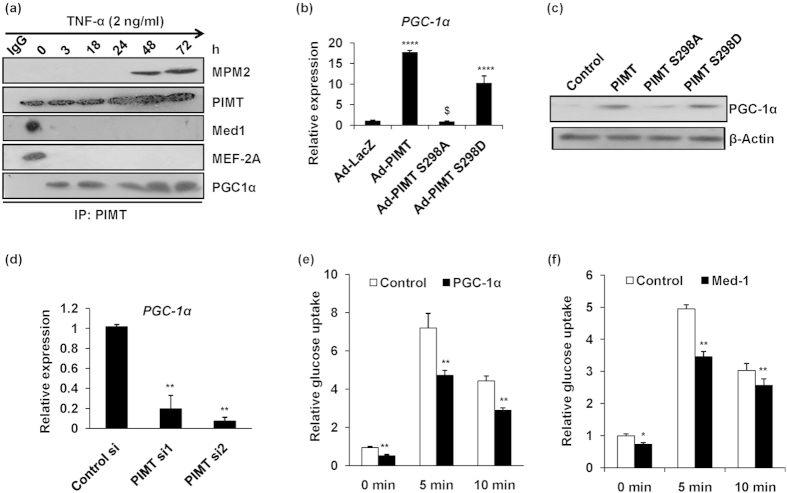
PIMT mediated inhibition of glucose uptake involves Med1 and PGC-1α. (**a**) Co-immunoprecipiation and western blotting to detect the phosphorylation of PIMT (MPM2), total PIMT, Med1, MEF2A and PGC-1α in TNF-α treated L6 myoblasts. The cropped blots were run under the same experimental conditions. The full-length blots are included in Supplemental Fig. 8. (**b**) mRNA levels of PGC-1α in Ad-PIMT and Ad-PIMT Ser^298^ mutant infected L6 myotubes. Values are shown as mean ± SD; ****p < 0.001 versus Ad-LacZ infected cells, ^$^*p* < 0.005 versus PIMT (WT) infected cells (two way ANOVA). (**c**) Western blotting for PGC-1α in WT and PIMT mutant transfected L6 myoblasts. The cropped blots were run under the same experimental conditions. The full-length blots are included in Supplemental Fig. 8. (**d**) mRNA levels of PGC-1α in PIMT siRNA transfected L6 myoblasts. Values are shown as mean ± SD; **p < 0.01 versus control transfected cells (two way ANOVA). (**e**,**f**) Basal and insulin stimulated (5 and 10 min) uptake of 2-NBDG by L6 myoblasts transfected with PGC-1α (**e**) and Med1 (**f**). Values are shown as mean ± SD after normalizing with the corresponding protein content and expressed relative to basal of control cells which was set to 1; **p < 0.001versus corresponding control cells (two way ANOVA).

We have previously showed that PIMT interacts with Med1^47^ and additively enhances the hepatic glucose output via the up-regulation of hepatic gluconeogenic genes such as PGC-1α in MEK/ERK dependent manner^49^. Moreover, muscle-specific Med1 knockout mice showed enhanced insulin sensitivity and improved glucose tolerance^61^. Thus, we wondered whether PIMT mediated inhibition of glucose uptake may involve Med1 and/or PGC-1α. To examine this, we studied the interaction between Med1 and PIMT in TNF-α stimulated L6 myoblasts (Fig. 7a). Interaction between Med1 and PIMT was evident in un-stimulated cells however their association was disrupted in TNF-α exposed L6 myoblasts. Similar trend was observed with MEF2A (Fig. 7a). The association of PIMT with PGC-1α was not detectable in un-stimulated cells, however PIMT-PGC-1α complex was readily observed upon TNF-α stimulation (Fig. 7a). Further, forced expression of WT PIMT or phospho-mimic mutant Ser298Asp robustly augmented PGC-1α levels (Fig. 7b,c and Supplemental Fig. 6d). Additionally, knockdown of PIMT dramatically suppressed PGC-1α transcript levels in L6 myoblasts (Fig. 7d). Having observed the interaction between PIMT and Med1/PGC-1α, we next tested their involvement in glucose uptake. Overexpression of Med1 or PGC-1α modestly suppressed insulin-stimulated glucose uptake (Fig. 7e,f) suggesting that Med1 and PGC-1α may contribute partly to PIMT mediated inhibition of glucose uptake.

### Ectopic expression of PIMT in the skeletal muscle of rats impaired the expression of GLUT4, MEF2 isoforms, HDAC5 and PGC-1α

**Figure 8:**
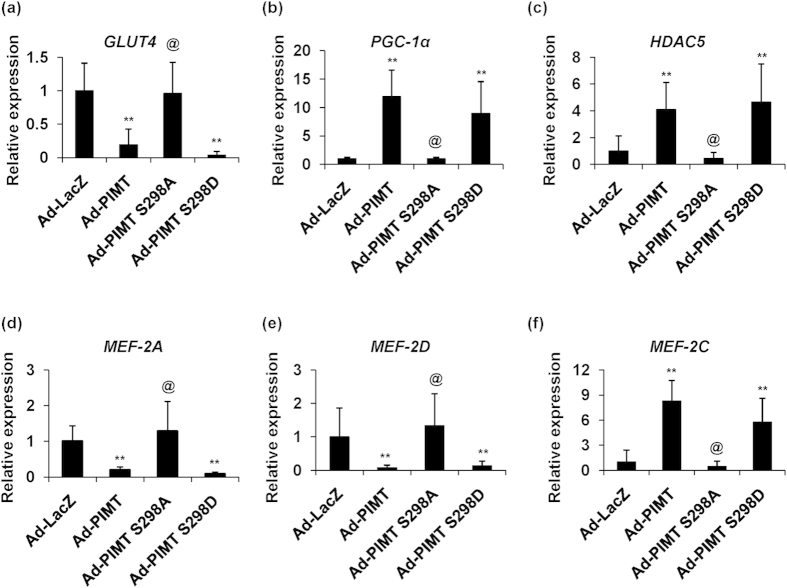
Overexpression of PIMT abrogates GLUT4 and MEF2A expression *in vivo* in Ser^298^ phosphorylation dependent manner. (**a**–**f**) mRNA expression of GLUT4 (**a**), PGC-1α (**b**), HDAC5 (**c**), MEF2A (**d**), MEF2D (**e**) and MEF2C (**f**) in Ad/PIMT (WT and mutant) injected rat skeletal muscle tissues. Values are shown as mean ± SD; **p < 0.01, versus Ad-LacZ injected rat skeletal muscle tissue, ^@^*p* < 0.01 versus Ad-PIMT infected rat skeletal muscle tissue (two way ANOVA).

Results from the *in vitro* experiments led us to investigate the impact of PIMT on GLUT4 and other genes in rat skeletal muscle by overexpressing PIMT in the right gastrocnemius skeletal muscle of Wistar rats as previously described^62^. Effectiveness of the procedure was confirmed by fluorescence in the processed skeletal muscle tissue infected with Ad-PIMT EGFP (Supplemental Fig. 7a). Infection of rat skeletal muscle with Ad-PIMT EGFP resulted in a robust decrease in the transcript levels of GLUT4, MEF-2A and MEF-2D (Fig. 8a,d,e). In contrast, the mRNA levels of PGC-1α, HDAC5 and MEF-2C were found to be elevated in Ad-PIMT EGFP infected skeletal muscle tissue (Fig. 8b,c,f). Consistent with the data from cultured cells, infection with Ad-PIMT Ser298Asp but not Ad-PIMT Ser298Ala suppressed GLUT4, MEF2A, MEF2D (Fig. 8a,d,e) expression while up-regulating PGC1-α, HDAC5 and MEF2C expression in the rat skeletal muscle tissue (Fig. 8b,c,f). qPCR analysis showed that the expression levels of human PIMT and Ser^298^ PIMT mutants were comparable in the skeletal muscle of the Wistar rats (Supplemental Fig. 7b).

Collectively, our data establish that the transcriptional co-activator binding protein, PIMT, mediates TNF-α induced insulin resistance in the skeletal muscle via the transcriptional modulation of several genes associated with glucose uptake.

## Discussion

Insulin resistance is an intrinsic defect of T2D developing several years before overt glycemia is observed^13^,^63^,^64^. Given that T2D patients characteristically show insulin resistance at skeletal muscle, the main organ for insulin mediated glucose disposal, it is crucial to understand the mechanisms of the insulin resistance development in this tissue^8^,^13^,^65^,^66^. It has become apparent over the last decade that the etiology of obesity-induced insulin resistance is complex and several independent studies demonstrate that chronic inflammation is strongly linked to the development and progression of insulin resistance^15^,^16^,^17^,^50^,^51^,^64^. Accumulation of macrophages, the principal architects of inflammation, was reported in the skeletal muscle of HFD fed mice and obese diabetics^67^. Moreover, pro-inflammatory cytokine, TNF-α is enhanced in the skeletal muscle and other tissues of humans and animals with insulin resistance and/or diabetes^17^,^18^,^19^,^20^,^21^,^22^,^24^,^39^,^51^,^53^,^68^,^69^,^70^. The underlying mechanisms of TNF-α mediated insulin resistance are vaguely understood. In the current study, we identified that the transcriptional co-activator binding protein, PIMT, is an important mediator of TNF-α induced skeletal muscle insulin resistance. We observed that 1) the expression of PIMT was enhanced in TNF-α exposed cultured skeletal muscle cells and soleus muscle of HSD fed rats 2) PIMT expression regulates insulin receptor signaling cascade 3) TNF-α stimulated phosphorylation of PIMT at Ser^298^ 4) PIMT suppressed insulin stimulated glucose uptake via the modulation of the expression of GLUT4, MEF2A and HDAC5 in cultured cells and skeletal muscle of rats in phosphorylation dependent manner. It was earlier reported that GLUT4 mRNA levels were almost undetectable 24 h post TNF-α incubation of human adipose cells, however the underlying mechanisms are unknown^38^. Based on our observations that i) TNF-α augmented PIMT protein levels 24 h and beyond ii) GLUT4 mRNA and protein are barely discernible in PIMT overexpressing myoblasts/myotubes, we propose that PIMT may be the major driver of the TNF-α mediated repression of GLUT4.

Apart from transcriptional repression of GLUT4, PIMT has also emerged as an important player in the regulation of insulin signaling. Post translational phospho-modification of IRS proteins induced by different stimuli control insulin signaling cascade. Extensive studies in different diabetic animal models (HFD, *ob/ob, db/db*) supported by cell based observations unambiguously established that chronic inflammation (TNF-α) activated JNK1/2 and p38 phosphorylate IRS1 at Ser^307^ position which inhibits insulin signaling leading to IR^71^,^72^. Our findings that PIMT disrupts insulin signaling potentially through the induction of TNF-α mediated phosphorylation of JNK and p38 suggests that PIMT may have a broader role in glucose control by skeletal muscle. The underlying mechanisms of PIMT effects on insulin signaling pathway are not clear at this stage. PIMT being a transcriptional co-activator binding protein, we do not envisage any direct role for PIMT in insulin signaling cascade. Given the effects of PIMT on the phosphorylation levels of JNK and p38, we speculate that PIMT may influence insulin signaling indirectly by the regulating the expression of genes linked to lipid metabolism^72^.

The mechanisms underlying the elevated expression of PIMT in TNF-α treated cultured cells and the muscle of HSD fed rats are unknown. Analysis of the upstream region of the PIMT gene showed multiple binding sites for the transcription factor, NFκB, which is well documented to be activated in skeletal muscle cells upon TNF-α stimulation^73^,^74^,^75^. Moreover, hyperinsulinemia observed in HSD fed rats may have also contributed to the increased expression of PIMT in the skeletal muscle. Supporting this possibility, we observed that the protein levels of PIMT were elevated in L6 myoblasts stimulated with insulin for 48 h (data not shown). In the current study, we have also observed that TNF-α induces phosphorylation of PIMT at Ser^298^, a site that we have previously characterized as an ERK2 target site^49^. Importantly, treatment of L6 cells with the MEK/ERK inhibitor, U0126, blunted the inhibitory effect of PIMT on the glucose uptake suggesting a negative regulatory role for ERK-mediated phosphorylation of PIMT in skeletal muscle insulin sensitivity. This inference about the functionality of ERK1/2 in insulin resistance is consistent with observations from several previous studies. The activity of ERK1/2 was shown to be increased in adipose tissue of diet induced obese mice^76^,^77^ and liver of genetically obese Zucker (*fa/fa*) rats^78^. Further, defective activation of ERK1/2 was strikingly observed in obesity-induced insulin resistant human skeletal muscle^79^. Furthermore, basal phosphorylation of ERK1/2 was elevated in skeletal muscle biopsies of insulin resistant women with polycystic ovary syndrome^80^,^81^. Moreover, activation of MEK1/ERK pathway by TNF-α suppressed tyrosine phosphorylation and enhanced serine phosphorylation of IRS1 in 3T3L1 adipocytes^82^. In addition, MEK1 mutant which activates ERK, markedly downregulated the expression of insulin receptor and its downstream substrates IRS1 and IRS2 in TNF-α treated 3T3L1 adipocytes^83^. Expectedly, genetic ablation or pharmacological inhibition of ERK1/2 activity ameliorated insulin sensitivity in various models by altering insulin dependent as well as insulin independent signaling (AMPK)^76^,^78^,^84^,^85^,^86^,^87^,^88^. Importantly, the finding that phosphorylation of PIMT at ERK-target site Ser^298^ impairs glucose uptake in skeletal muscle is conceptually in agreement with our previous finding that the phosphorylation of the same residue is critical for PIMT mediated augmentation of hepatic gluconeogenesis suggesting an inhibitory role for ERK-mediated phosphorylation of PIMT in glucose metabolism. Further studies should be performed to delineate the significance of PIMT phosphorylation in other glucose homeostatic mechanisms.

The mechanisms through which PIMT suppresses glucose uptake in un-stimulated and TNF-α exposed myoblasts appear to be strikingly different. In un-stimulated cells, PIMT was observed to interact with MEF-2A and Med1. PIMT is potentially recruited to MEF-2A response element of GLUT4 promoter through its association with MEF-2A. Since PIMT was associated with GLUT4 promoter in un-stimulated cells, it is possible that PIMT is poised to repress basal transcription of GLUT4. Med1 may be another component of this transcriptional repressor complex. Supporting this, overexpression of Med1 partially inhibited insulin stimulated glucose uptake by L6 myoblasts, a finding in concordance with the phenotype of muscle specific Med1 knockout mice which displayed improved glucose tolerance, enhanced insulin sensitivity and resistance to high fat induced obesity^61^. However, the association between PIMT and MEF-2A/Med1 seems to be disrupted in TNF-α stimulated cells and PIMT was observed to bind to PGC-1α upon stimulation. In our previous study we failed to observe any physical association between PIMT and PGC-1α^47^ *in vitro*. However, in the current study we observed that PIMT and PGC-1α are together in a molecular complex in TNF-α stimulated L6 cells. This suggests that the interaction between PIMT and PGC-α is context specific and probably indirect. Consistent with our previous study^49^, we observed that forced expression of PIMT in cultured myoblasts or rat skeletal muscle enhanced the expression levels of PGC-1α indicating that PIMT controls the expression of PGC-1α in both liver and skeletal muscle. Our data are suggestive of an inhibitory role for PGC-1α in muscle insulin sensitivity; however, the functional role of PGC-1α in muscle insulin sensitivity remains ambiguous even after more than a decade of the first study^89^. Initial *in vitro* studies showed that PGC-1α boosts glucose uptake in L6 and C2C12 myotubes via the up-regulation of GLUT4 expression^90^. On the contrary, a subsequent study showed that muscle specific PGC-1α transgenic mice were more prone to high fat diet induced insulin resistance due to diminished insulin stimulated glucose uptake^91^. Further, data from muscle specific knockout mice of both PGC-1α and PGC-1β reveal that the co-activators are required for oxidative and mitochondrial programs of skeletal muscle but are dispensable for insulin sensitivity^92^. Thus, additional studies are warranted to determine the precise role of PGC-1α in controlling muscle insulin sensitivity.

In summary, we have shown that the expression of PIMT is up-regulated in TNF-α treated myoblasts and skeletal muscle of HSD fed rats. TNF-α stimulation induced phosphorylation of PIMT at an ERK2 target site. Further, we provide evidence that PIMT blunts insulin stimulated glucose uptake through i) the modulation of the expression of GLUT4, MEF2A and HDAC5 in phosphorylation dependent manner ii) abrogation of insulin signaling. Thus, our current and previous findings^49^ position PIMT as a novel therapeutic target for T2D and given the significance of the ERK-mediated Ser^298^ phosphorylation of PIMT in glucose metabolism, strategies may be designed to specifically inhibit ERK-mediated PIMT phosphorylation for improved glucose homeostasis.

## Methods

### Expression and reporter constructs

Expression constructs pCMV-PIMT-Flag, pcDNA3.1-PIMT^S298A^, pcDNA3.1-PIMT^S298D^, pcDNA3.1-PGC-1α and pCMX-Med1 were described previously^47^,^49^,^93^. Wild type GLUT4.Luc.779 and MEF2 binding defective GLUT4.Luc.MEF-Mut were generously gifted by Dr. Brian N. Finck, Washington University, St. Louis^94^.

### Animal treatment

All the methods were carried out in accordance with the approved guidelines. Colony bred healthy adult Wistar albino rats, weighing 150 ± 10 g were maintained in polypropylene cages in a standard photoperiod (12 h light:12 h dark cycle) and temperature (27 ± 1 °C) controlled room with the provision of laboratory food (Gold Mohur feeds Ltd, New Delhi, India) and water *ad libitum*. Rats were given standard chow diet (control group) or high sucrose diet (65%, treated group) for a period of 60 days. Post treatment animals were sacrificed in accordance with the approved guidelines of the Committee for the Purpose of Control and Supervision on Experiments on Animals (CPCSEA). Experimental protocol involving animals was reviewed and approved by the Animal Ethical Committee of Institute of Science, Nirma University, Ahmedabad, India (Protocol No. IS/BT/FAC-13-1009).

### OGTT

Oral glucose tolerance test (OGTT) was performed on overnight fasted rats as described earlier^95^. Animals were orally injected with 20% glucose solution (2 g/kg). Blood was collected at 0, 15, 60, 90 and 120 min. Glucose concentrations were determined with a Free Style Optium H Blood Glucose Monitor (Abbott, Maidenhead, UK).

### Estimation of insulin and triglyceride

Serum insulin level was assayed by rat insulin ELISA kit (Mercodia, Uppsala, Sweden). The plasma triglyceride was measured using a diagnostic kit from Accucare India Ltd.

### TNF-α measurement

Levels of TNF-α in sera collected from various animal groups were estimated using Multiplex CBA Rat Inflammation kit (BD Biosciences, CA, USA). Briefly, different capture bead populations were mixed, incubated with protein standards or test samples, and subsequently incubated with PE-conjugated detection antibodies (measured in FL2) to form sandwich complexes. The standard and test samples were analyzed using CBA software in flow cytometric platform.

### Cell culture, transfection and adenovirus infection

L6 cells and HEK293T were maintained in Dulbecco’s Modified Eagle’s Medium (DMEM) supplemented with 10% FBS at 37 °C with 5% CO_2_. L6 myoblasts were cultured to confluence and the medium was switched to differentiation medium (DMEM with 2% horse serum), which was changed every alternate day. After 4 additional days, the differentiated L6 cells fused to form myotubes. Post differentiation, myotubes were infected with adenovirus at a multiplicity of infection of 300 plaque forming units per cell. Ad/PIMT eGFP, Ad/PIMT^S298A^, Ad/PIMT^S298D^ and Ad/LacZ were described previously^49^. The subsequent analysis was performed post 48 h of infection. The Rat *PIMT* specific siRNAs were procured from Dharmacon (Thermo-Scientific, PA, USA) and control siRNA (siCONTROL) was obtained from Ambion (Invitrogen, CA, USA). Transient transfections with all expression constructs and siRNAs were performed using Lipofectamine^TM^ 2000 (Invitrogen, CA, USA) as per manufacturer’s instructions. The final concentration of siRNA was 30nM. The cells transfected with expression constructs or siRNA were used for downstream experiments post 24 h or 48 h. Where indicated cells were stimulated with 2 ng/ml TNF-α (R&D Systems, MN, USA) for different time points as mentioned in the corresponding figure legends. Medium was changed every 24 h with fresh complete medium containing TNF-α.

### Isolation of neonatal rat skeletal myoblasts

Muscle tissue from the lower hind limbs of 3 day-old neonatal Wistar rats was dissected and minced. The minced tissue was digested in solution containing 0.07% collagenase, 0.35% trypsin and 0.2% glucose prepared in PBS for 30 min at 37 °C. The debris was removed by centrifugation at 50 g for 5 min at RT. Supernatant was collected and the enzymatic activity was neutralized by the addition of 100 μL of FBS. The cells were counted, re-suspended in growth medium (DMEM, 10% v/v FBS, 100 units/ml Penicillin-G, 100 μg/ml Streptomycin, 0.1 mM β-mercaptoethanol) and plated in collagen coated cell binding plates for 2 h at 37 °C to enrich for the myoblast population.

### 2-NBDG glucose uptake

2-(N-[7-nitrobenz-2-oxa-1,3-diazol-4-yl]amino)-2-deoxyglucose (2-NBDG) (Molecular Probes-Invitrogen, CA , USA) was used to assess glucose uptake in L6 and neonatal cells. Approximately 20,000 cells/well were seeded in a 24 well-plate, transfected/infected and/or stimulated with TNF-α where indicated followed by serum starvation overnight. Cells were kept in glucose free medium for 30 min prior to 100 nM insulin stimulation for 5 and 10 min respectively. Insulin primed cells were incubated with 50 μM of 2-NBDG for 15 min. Reaction was stopped by washing cells with ice cold 1X PBS and the cells were lysed in 0.1% Triton X-100. The fluorescence intensity of the lysates was measured on a Wallac 1420 Victor Multimode Plate Reader (Perkin-Elmer, MA, USA) with excitation at 485 nm and emission at 535 nm.

### Western blotting

Immunoblotting was performed as described earlier^49^ using anti-PIMT (1:1000; Santa Cruz Biotechnology, CA, USA) anti-GLUT4 (1:1000; Novus Biologicals, MO, USA), anti-MEF2A (1:1000; Cell Signaling Technology, MA, USA), anti-PGC-1 (1:1000; Santa Cruz Biotechnology, CA, USA),anti-pSer307-IRS1 (1:1000; Cell Signaling Technology, MA, USA),anti-pTyr608/612-IRS (1:1000; Abcam, MA, USA), anti-IRS1 (1:1000; Cell Signaling Technology, MA, USA), anti-pJNK1/2 (1:1000; Cell Signaling Technology, MA, USA), anti-pAkt (Ser473) (1:1000; Cell Signaling Technology, MA, USA), anti-Akt (1:1000; Cell Signaling Technology, MA, USA) ,anti-ERK1/2 (1:1000; Cell Signaling Technology, MA, USA) and anti-pERK1/2 (1:1000; Cell Signaling Technology, MA, USA) using specific antibodies and appropriate HRP-conjugated secondary antibody. Membranes were stripped and re-probed with β-actin (1:1000, ICN Biomedicals Inc., CA, USA) and Tubulin (1:1000, Genscript, NJ, USA) as the protein loading control.

### Immumoprecipitation and Chromatin Immumoprecipitation

Cells were scraped, cellular proteins lysates were prepared using lysis buffer [120 mM NaCl, 1% Triton X-100, 20 mM Tris–HCl pH 7.5, 10% glycerol, 2 mM EDTA and protease inhibitor cocktail (Roche GmbH, Germany)]. For immunopreciptition, 4 mg of protein lysate per sample was immunoprecipited using anti-PIMT (2 μg; Santacruz Biotechnology, CA ,USA) and was resolved on to 10% SDS–polyacrylamide gel, probed by anti-MPM2 (ERK1/2 substrate antibody; Millipore, MA, USA) followed by reprobed with PIMT, Med-1 (1:1000; Santacruz Biotechnologies, CA, USA), PGC-1α and MEF2A specific antibodies and appropriate HRP-conjugated secondary antibody. ChIP was performed as described earlier^49^.

### Real-Time PCR

For qPCR, reverse transcription was performed with 4 μg of total RNA using random hexamer in a reaction volume of 20 μl using Superscript III First Strand cDNA Synthesis kit (Invitrogen, CA, USA). Quantitative PCR for *PIMT, GLUT4, MEF2A, MEF2D, MEF2C, PGC-1α and HDAC5* genes were performed using SYBR Green master mix (Invitrogen, CA, USA) on master cycler ABI, Step-One Plus (Invitrogen, CA, USA). The mRNA expression was normalized to 18S rRNA with the values for control arbitrarily set to 1. All the experiments were repeated three times.

### Luciferase reporter assay

For GLUT4 promoter activity studies, HEK 293T cells were co-transfected with GLUT4.Luc and GLUT4.Luc.MEF-Mut promoter in addition to other expression constructs as mentioned in figure. Each transfection mix contained 400 ng of reporter construct, 100 ng of renilla luciferase expression vector along with either of 300 ng of expression constructs. Cells were lysed 24 h after transfection and lysates were analyzed as described previously^49^.

### Adenoviral injection into rat skeletal muscle

Adenovirus injection in muscle was performed as described by Quantin *et al.*^62^. Briefly, the recombinant virus (10^8^ plaque forming units) was injected directly into the right skeletal muscle. Post 5 days of injection, animals were sacrificed and the muscle tissue (right muscle) was collected for further analysis.

### Statistical Analysis

Values were expressed as mean ± S.D. For comparison between 2 groups, the unpaired Student’s *t*-test was used. Two-way ANOVA followed by Bonferroni’s post hoc analysis was used to compare more than 2 groups. *p* < 0.05 was considered as significant.

## Additional Information

**How to cite this article**: Kain, V. *et al.* Co-activator binding protein PIMT mediates TNF-a induced insulin resistance in skeletal muscle via the transcriptional down-regulation of MEF2A and GLUT4. *Sci. Rep.* **5**, 15197; doi: 10.1038/srep15197 (2015).

## Supplementary Material

Supplementary Information

## References

[b1] Wilcox G. Insulin and insulin resistance. Clin Biochem Rev 26, 19–39 (2005). 16278749 PMC1204764

[b2] Schinner S., Scherbaum W. A., Bornstein S. R. & Barthel A. Molecular mechanisms of insulin resistance. Diabet Med 22, 674–682, 10.1111/j.1464-5491.2005.01566.x (2005). 15910615

[b3] Bhattacharya S., Dey D. & Roy S. S. Molecular mechanism of insulin resistance. J Biosci 32, 405–413 (2007). 17435330 10.1007/s12038-007-0038-8

[b4] Schaefer E. J., Gleason J. A. & Dansinger M. L. Dietary fructose and glucose differentially affect lipid and glucose homeostasis. J Nutr 139, 1257S–1262S, 10.3945/jn.108.098186 (2009). 19403705 PMC2682989

[b5] Basciano H., Federico L. & Adeli K. Fructose, insulin resistance, and metabolic dyslipidemia. Nutr Metab (Lond) 2, 5, 10.1186/1743-7075-2-5 (2005). 15723702 PMC552336

[b6] Alwahsh S. M. *et al.* Combination of alcohol and fructose exacerbates metabolic imbalance in terms of hepatic damage, dyslipidemia, and insulin resistance in rats. PLoS One 9, e104220, 10.1371/journal.pone.0104220 (2014). 25101998 PMC4125190

[b7] Parks B. W. *et al.* Genetic control of obesity and gut microbiota composition in response to high-fat, high-sucrose diet in mice. Cell Metab 17, 141–152, 10.1016/j.cmet.2012.12.007 (2013). 23312289 PMC3545283

[b8] Abdul-Ghani M. A. & DeFronzo R. A. Pathogenesis of insulin resistance in skeletal muscle. J Biomed Biotechnol 2010, 476279, 10.1155/2010/476279 (2010). 20445742 PMC2860140

[b9] Hribal M. L., Oriente F. & Accili D. Mouse models of insulin resistance. Am J Physiol Endocrinol Metab 282, E977–981, 10.1152/ajpendo.00561.2001 (2002). 11934661

[b10] Kim J. A., Montagnani M., Koh K. K. & Quon M. J. Reciprocal relationships between insulin resistance and endothelial dysfunction: molecular and pathophysiological mechanisms. Circulation 113, 1888–1904, 10.1161/CIRCULATIONAHA.105.563213 (2006). 16618833

[b11] Ferrannini E. *et al.* Insulin: new roles for an ancient hormone. Eur J Clin Invest 29, 842–852 (1999). 10583426 10.1046/j.1365-2362.1999.00536.x

[b12] Cline G. W. *et al.* Impaired glucose transport as a cause of decreased insulin-stimulated muscle glycogen synthesis in type 2 diabetes. N Engl J Med 341, 240–246, 10.1056/NEJM199907223410404 (1999). 10413736

[b13] DeFronzo R. A. & Tripathy D. Skeletal muscle insulin resistance is the primary defect in type 2 diabetes. Diabetes Care 32 Suppl 2, S157–163, 10.2337/dc09-S302 (2009). 19875544 PMC2811436

[b14] Boden G. Obesity and free fatty acids. Endocrinol Metab Clin North Am 37, 635–646, viii-ix, 10.1016/j.ecl.2008.06.007 (2008). 18775356 PMC2596919

[b15] Shoelson S. E., Lee J. & Goldfine A. B. Inflammation and insulin resistance. J Clin Invest 116, 1793–1801, 10.1172/JCI29069 (2006). 16823477 PMC1483173

[b16] Olefsky J. M. & Glass C. K. Macrophages, inflammation, and insulin resistance. Annu Rev Physiol 72, 219–246, 10.1146/annurev-physiol-021909-135846 (2010). 20148674

[b17] Dandona P., Aljada A. & Bandyopadhyay A. Inflammation: the link between insulin resistance, obesity and diabetes. Trends Immunol 25, 4–7 (2004). 14698276 10.1016/j.it.2003.10.013

[b18] Uysal K. T., Wiesbrock S. M., Marino M. W. & Hotamisligil G. S. Protection from obesity-induced insulin resistance in mice lacking TNF-alpha function. Nature 389, 610–614, 10.1038/39335 (1997). 9335502

[b19] Hotamisligil G. S., Shargill N. S. & Spiegelman B. M. Adipose expression of tumor necrosis factor-alpha: direct role in obesity-linked insulin resistance. Science 259, 87–91 (1993). 7678183 10.1126/science.7678183

[b20] Kern P. A. *et al.* The expression of tumor necrosis factor in human adipose tissue. Regulation by obesity, weight loss, and relationship to lipoprotein lipase. J Clin Invest 95, 2111–2119, 10.1172/JCI117899 (1995). 7738178 PMC295809

[b21] Dandona P. *et al.* Tumor necrosis factor-alpha in sera of obese patients: fall with weight loss. J Clin Endocrinol Metab 83, 2907–2910, 10.1210/jcem.83.8.5026 (1998). 9709967

[b22] Saghizadeh M., Ong J. M., Garvey W. T., Henry R. R. & Kern P. A. The expression of TNF alpha by human muscle. Relationship to insulin resistance. J Clin Invest 97, 1111–1116, 10.1172/JCI118504 (1996). 8613535 PMC507159

[b23] Mingrone G. *et al.* Skeletal muscle triglycerides lowering is associated with net improvement of insulin sensitivity, TNF-alpha reduction and GLUT4 expression enhancement. Int J Obes Relat Metab Disord 26, 1165–1172, 10.1038/sj.ijo.0802053 (2002). 12187392

[b24] Hotamisligil G. S. The role of TNFalpha and TNF receptors in obesity and insulin resistance. J Intern Med 245, 621–625 (1999). 10395191 10.1046/j.1365-2796.1999.00490.x

[b25] Nieto-Vazquez I. *et al.* Insulin resistance associated to obesity: the link TNF-alpha. Arch Physiol Biochem 114, 183–194, 10.1080/13813450802181047 (2008). 18629684

[b26] Hotamisligil G. S. Mechanisms of TNF-alpha-induced insulin resistance. Exp Clin Endocrinol Diabetes 107, 119–125, 10.1055/s-0029-1212086 (1999). 10320052

[b27] Nguyen M. T. *et al.* JNK and tumor necrosis factor-alpha mediate free fatty acid-induced insulin resistance in 3T3-L1 adipocytes. J Biol Chem 280, 35361–35371, 10.1074/jbc.M504611200 (2005). 16085647

[b28] de Alvaro C., Teruel T., Hernandez R. & Lorenzo M. Tumor necrosis factor alpha produces insulin resistance in skeletal muscle by activation of inhibitor kappaB kinase in a p38 MAPK-dependent manner. J Biol Chem 279, 17070–17078, 10.1074/jbc.M312021200 (2004). 14764603

[b29] Xin-Long C., Zhao-Fan X., Dao-Feng B. & Wei D. mTOR partly mediates insulin resistance by phosphorylation of insulin receptor substrate-1 on serine(307) residues after burn. Burns 37, 86–93, 10.1016/j.burns.2010.04.005 (2011). 20594757

[b30] Bae E. J., Yang Y. M., Kim J. W. & Kim S. G. Identification of a novel class of dithiolethiones that prevent hepatic insulin resistance via the adenosine monophosphate-activated protein kinase-p70 ribosomal S6 kinase-1 pathway. Hepatology 46, 730–739, 10.1002/hep.21769 (2007). 17668885

[b31] Rui L. *et al.* Insulin/IGF-1 and TNF-alpha stimulate phosphorylation of IRS-1 at inhibitory Ser307 via distinct pathways. J Clin Invest 107, 181–189, 10.1172/JCI10934 (2001). 11160134 PMC199174

[b32] Kanety H., Feinstein R., Papa M. Z., Hemi R. & Karasik A. Tumor necrosis factor alpha-induced phosphorylation of insulin receptor substrate-1 (IRS-1). Possible mechanism for suppression of insulin-stimulated tyrosine phosphorylation of IRS-1. J Biol Chem 270, 23780–23784 (1995). 7559552 10.1074/jbc.270.40.23780

[b33] Ruan H., Hacohen N., Golub T. R., Van Parijs L. & Lodish H. F. Tumor necrosis factor-alpha suppresses adipocyte-specific genes and activates expression of preadipocyte genes in 3T3-L1 adipocytes: nuclear factor-kappaB activation by TNF-alpha is obligatory. Diabetes 51, 1319–1336 (2002). 11978627 10.2337/diabetes.51.5.1319

[b34] Steinberg G. R. *et al.* Tumor necrosis factor alpha-induced skeletal muscle insulin resistance involves suppression of AMP-kinase signaling. Cell Metab 4, 465–474, 10.1016/j.cmet.2006.11.005 (2006). 17141630

[b35] Ishizuka K. *et al.* Chronic tumor necrosis factor-alpha treatment causes insulin resistance via insulin receptor substrate-1 serine phosphorylation and suppressor of cytokine signaling-3 induction in 3T3-L1 adipocytes. Endocrinology 148, 2994–3003, 10.1210/en.2006-1702 (2007). 17379643

[b36] Rotter V., Nagaev I. & Smith U. Interleukin-6 (IL-6) induces insulin resistance in 3T3-L1 adipocytes and is, like IL-8 and tumor necrosis factor-alpha, overexpressed in human fat cells from insulin-resistant subjects. J Biol Chem 278, 45777–45784, 10.1074/jbc.M301977200 (2003). 12952969

[b37] Sarvas J. L., Khaper N. & Lees S. J. The IL-6 Paradox: Context Dependent Interplay of SOCS3 and AMPK. J Diabetes Metab Suppl 13, 10.4172/2155-6156.S13-003 (2013). PMC3828628 24244888

[b38] Hauner H., Petruschke T., Russ M., Rohrig K. & Eckel J. Effects of tumour necrosis factor alpha (TNF alpha) on glucose transport and lipid metabolism of newly-differentiated human fat cells in cell culture. Diabetologia 38, 764–771 (1995). 7556976 10.1007/s001250050350

[b39] Stephens J. M., Lee J. & Pilch P. F. Tumor necrosis factor-alpha-induced insulin resistance in 3T3-L1 adipocytes is accompanied by a loss of insulin receptor substrate-1 and GLUT4 expression without a loss of insulin receptor-mediated signal transduction. J Biol Chem 272, 971–976 (1997). 8995390 10.1074/jbc.272.2.971

[b40] Morgan C. L., Puelles J., Poole C. D. & Currie C. J. The effect of withdrawal of rosiglitazone on treatment pathways, diabetes control and patient outcomes: a retrospective cohort study. J Diabetes Complications 28, 360–364, 10.1016/j.jdiacomp.2014.01.007 (2014). 24529918

[b41] Zhu Y. *et al.* Cloning and characterization of PIMT, a protein with a methyltransferase domain, which interacts with and enhances nuclear receptor coactivator PRIP function. Proc Natl Acad Sci USA 98, 10380–10385, 10.1073/pnas.181347498 (2001). 11517327 PMC56969

[b42] Monecke T., Dickmanns A. & Ficner R. Structural basis for m7G-cap hypermethylation of small nuclear, small nucleolar and telomerase RNA by the dimethyltransferase TGS1. Nucleic Acids Res 37, 3865–3877, 10.1093/nar/gkp249 (2009). 19386620 PMC2709555

[b43] Franke J., Gehlen J. & Ehrenhofer-Murray A. E. Hypermethylation of yeast telomerase RNA by the snRNA and snoRNA methyltransferase Tgs1. J Cell Sci 121, 3553–3560, 10.1242/jcs.033308 (2008). 18840651

[b44] Hausmann S. *et al.* Genetic and biochemical analysis of yeast and human cap trimethylguanosine synthase: functional overlap of 2,2,7-trimethylguanosine caps, small nuclear ribonucleoprotein components, pre-mRNA splicing factors, and RNA decay pathways. J Biol Chem 283, 31706–31718, 10.1074/jbc.M806127200 (2008). 18775984 PMC2581544

[b45] Tang W., Kannan R., Blanchette M. & Baumann P. Telomerase RNA biogenesis involves sequential binding by Sm and Lsm complexes. Nature 484, 260–264, 10.1038/nature10924 (2012). 22446625 PMC3326189

[b46] Wurth L. *et al.* Hypermethylated-capped selenoprotein mRNAs in mammals. Nucleic Acids Res 42, 8663–8677, 10.1093/nar/gku580 (2014). 25013170 PMC4117793

[b47] Misra P. *et al.* Interaction of PIMT with transcriptional coactivators CBP, p300, and PBP differential role in transcriptional regulation. J Biol Chem 277, 20011–20019, 10.1074/jbc.M201739200 (2002). 11912212

[b48] Jia Y. *et al.* Early embryonic lethality of mice with disrupted transcription cofactor PIMT/NCOA6IP/Tgs1 gene. Mech Dev 129, 193–207, 10.1016/j.mod.2012.08.002 (2012). 22982455 PMC3503541

[b49] Kapadia B. *et al.* ERK2-mediated phosphorylation of transcriptional coactivator binding protein PIMT/NCoA6IP at Ser298 augments hepatic gluconeogenesis. PLoS One 8, e83787, 10.1371/journal.pone.0083787 (2013). 24358311 PMC3866170

[b50] Martins A. R. *et al.* Mechanisms underlying skeletal muscle insulin resistance induced by fatty acids: importance of the mitochondrial function. Lipids Health Dis 11, 30, 10.1186/1476-511X-11-30 (2012). 22360800 PMC3312873

[b51] Plomgaard P. *et al.* Tumor necrosis factor-alpha induces skeletal muscle insulin resistance in healthy human subjects via inhibition of Akt substrate 160 phosphorylation. Diabetes 54, 2939–2945 (2005). 16186396 10.2337/diabetes.54.10.2939

[b52] Bouzakri K. & Zierath J. R. MAP4K4 gene silencing in human skeletal muscle prevents tumor necrosis factor-alpha-induced insulin resistance. J Biol Chem 282, 7783–7789, 10.1074/jbc.M608602200 (2007). 17227768

[b53] Xie L., Ortega M. T., Mora S. & Chapes S. K. Interactive changes between macrophages and adipocytes. Clin Vaccine Immunol 17, 651–659, 10.1128/CVI.00494-09 (2010). 20164250 PMC2849320

[b54] Nagaev I., Bokarewa M., Tarkowski A. & Smith U. Human resistin is a systemic immune-derived proinflammatory cytokine targeting both leukocytes and adipocytes. PLoS One 1, e31, 10.1371/journal.pone.0000031 (2006). 17183659 PMC1762367

[b55] Oshel K. M., Knight J. B., Cao K. T., Thai M. V. & Olson A. L. Identification of a 30-base pair regulatory element and novel DNA binding protein that regulates the human GLUT4 promoter in transgenic mice. J Biol Chem 275, 23666–23673, 10.1074/jbc.M001452200 (2000). 10825161

[b56] Mora S. & Pessin J. E. The MEF2A isoform is required for striated muscle-specific expression of the insulin-responsive GLUT4 glucose transporter. J Biol Chem 275, 16323–16328, 10.1074/jbc.M910259199 (2000). 10748204

[b57] Sparling D. P., Griesel B. A., Weems J. & Olson A. L. GLUT4 enhancer factor (GEF) interacts with MEF2A and HDAC5 to regulate the GLUT4 promoter in adipocytes. J Biol Chem 283, 7429–7437, 10.1074/jbc.M800481200 (2008). 18216015 PMC2276327

[b58] Ginnan R., Sun L. Y., Schwarz J. J. & Singer H. A. MEF2 is regulated by CaMKIIdelta2 and a HDAC4-HDAC5 heterodimer in vascular smooth muscle cells. Biochem J 444, 105–114, 10.1042/BJ20120152 (2012). 22360269 PMC3632366

[b59] Lu J., McKinsey T. A., Nicol R. L. & Olson E. N. Signal-dependent activation of the MEF2 transcription factor by dissociation from histone deacetylases. Proc Natl Acad Sci USA 97, 4070–4075, 10.1073/pnas.080064097 (2000). 10737771 PMC18151

[b60] McGee S. L. *et al.* AMP-activated protein kinase regulates GLUT4 transcription by phosphorylating histone deacetylase 5. Diabetes 57, 860–867, 10.2337/db07-0843 (2008). 18184930

[b61] Chen W., Zhang X., Birsoy K. & Roeder R. G. A muscle-specific knockout implicates nuclear receptor coactivator MED1 in the regulation of glucose and energy metabolism. Proc Natl Acad Sci USA 107, 10196–10201, 10.1073/pnas.1005626107 (2010). 20479251 PMC2890439

[b62] Quantin B., Perricaudet L. D., Tajbakhsh S. & Mandel J. L. Adenovirus as an expression vector in muscle cells *in vivo*. Proc Natl Acad Sci USA 89, 2581–2584 (1992). 1557362 10.1073/pnas.89.7.2581 PMC48705

[b63] Xu J. & Zou M. H. Molecular insights and therapeutic targets for diabetic endothelial dysfunction. Circulation 120, 1266–1286, 10.1161/CIRCULATIONAHA.108.835223 (2009). 19786641 PMC2910587

[b64] Caballero A. E. Endothelial dysfunction, inflammation, and insulin resistance: a focus on subjects at risk for type 2 diabetes. Curr Diab Rep 4, 237–246 (2004). 15265464 10.1007/s11892-004-0074-9

[b65] Peppa M., Koliaki C., Nikolopoulos P. & Raptis S. A. Skeletal muscle insulin resistance in endocrine disease. J Biomed Biotechnol 2010, 527850, 10.1155/2010/527850 (2010). 20300436 PMC2840413

[b66] DeFronzo R. A. *et al.* The effect of insulin on the disposal of intravenous glucose. Results from indirect calorimetry and hepatic and femoral venous catheterization. Diabetes 30, 1000–1007 (1981). 7030826 10.2337/diab.30.12.1000

[b67] Fink L. N. *et al.* Pro-Inflammatory macrophages increase in skeletal muscle of high fat-Fed mice and correlate with metabolic risk markers in humans. Obesity (Silver Spring) 22, 747–757, 10.1002/oby.20615 (2014). 24030890

[b68] Alexander J. P. & Acott T. S. Involvement of the Erk-MAP kinase pathway in TNFalpha regulation of trabecular matrix metalloproteinases and TIMPs. Invest Ophthalmol Vis Sci 44, 164–169 (2003). 12506070 10.1167/iovs.01-1201

[b69] Barbin G., Roisin M. P. & Zalc B. Tumor necrosis factor alpha activates the phosphorylation of ERK, SAPK/JNK, and P38 kinase in primary cultures of neurons. Neurochem Res 26, 107–112 (2001). 11478736 10.1023/a:1011086426652

[b70] Fernandez-Veledo S. *et al.* Ceramide mediates TNF-alpha-induced insulin resistance on GLUT4 gene expression in brown adipocytes. Arch Physiol Biochem 112, 13–22, 10.1080/13813450500508137 (2006). 16754199

[b71] Johnson A. M. & Olefsky J. M. The origins and drivers of insulin resistance. Cell 152, 673–684, 10.1016/j.cell.2013.01.041 (2013). 23415219

[b72] Qatanani M. & Lazar M. A. Mechanisms of obesity-associated insulin resistance: many choices on the menu. Genes Dev 21, 1443–1455, 10.1101/gad.1550907 (2007). 17575046

[b73] Li Y. P., Schwartz R. J., Waddell I. D., Holloway B. R. & Reid M. B. Skeletal muscle myocytes undergo protein loss and reactive oxygen-mediated NF-kappaB activation in response to tumor necrosis factor alpha. FASEB J 12, 871–880 (1998). 9657527 10.1096/fasebj.12.10.971

[b74] Reid M. B. & Li Y. P. Tumor necrosis factor-alpha and muscle wasting: a cellular perspective. Respir Res 2, 269–272 (2001). 11686894 10.1186/rr67 PMC59514

[b75] Li Y. P. & Reid M. B. NF-kappaB mediates the protein loss induced by TNF-alpha in differentiated skeletal muscle myotubes. Am J Physiol Regul Integr Comp Physiol 279, R1165–1170 (2000). 11003979 10.1152/ajpregu.2000.279.4.R1165

[b76] Banks A. S. *et al.* An ERK/Cdk5 axis controls the diabetogenic actions of PPARgamma. Nature, 10.1038/nature13887 (2014). PMC4297557 25409143

[b77] Bost F., Aouadi M., Caron L. & Binetruy B. The role of MAPKs in adipocyte differentiation and obesity. Biochimie 87, 51–56, 10.1016/j.biochi.2004.10.018 (2005). 15733737

[b78] Zheng Y. *et al.* Improved insulin sensitivity by calorie restriction is associated with reduction of ERK and p70S6K activities in the liver of obese Zucker rats. J Endocrinol 203, 337–347, 10.1677/JOE-09-0181 (2009). 19801385 PMC3050029

[b79] Ruiz-Alcaraz A. J. *et al.* Obesity-induced insulin resistance in human skeletal muscle is characterised by defective activation of p42/p44 MAP kinase. PLoS One 8, e56928, 10.1371/journal.pone.0056928 (2013). 23468892 PMC3585240

[b80] Corbould A., Zhao H., Mirzoeva S., Aird F. & Dunaif A. Enhanced mitogenic signaling in skeletal muscle of women with polycystic ovary syndrome. Diabetes 55, 751–759 (2006). 16505239 10.2337/diabetes.55.03.06.db05-0453

[b81] Rajkhowa M. *et al.* Insulin resistance in polycystic ovary syndrome is associated with defective regulation of ERK1/2 by insulin in skeletal muscle *in vivo*. Biochem J 418, 665–671, 10.1042/BJ20082176 (2009). 19053948

[b82] De Fea K. & Roth R. A. Modulation of insulin receptor substrate-1 tyrosine phosphorylation and function by mitogen-activated protein kinase. J Biol Chem 272, 31400–31406 (1997). 9395471 10.1074/jbc.272.50.31400

[b83] Fujishiro M. *et al.* Three mitogen-activated protein kinases inhibit insulin signaling by different mechanisms in 3T3-L1 adipocytes. Mol Endocrinol 17, 487–497, 10.1210/me.2002-0131 (2003). 12554784

[b84] Jiao P., Feng B., Li Y., He Q. & Xu H. Hepatic ERK activity plays a role in energy metabolism. Mol Cell Endocrinol 375, 157–166, 10.1016/j.mce.2013.05.021 (2013). 23732116 PMC3733366

[b85] Liu H. *et al.* Hepatic serum- and glucocorticoid-regulated protein kinase 1 (SGK1) regulates insulin sensitivity in mice via extracellular-signal-regulated kinase 1/2 (ERK1/2). Biochem J 464, 281–289, 10.1042/BJ20141005 (2014). 25222560

[b86] Bi L. *et al.* Saturated fatty acids activate ERK signaling to downregulate hepatic sortilin 1 in obese and diabetic mice. J Lipid Res 54, 2754–2762, 10.1194/jlr.M039347 (2013). 23904453 PMC3770088

[b87] Hwang S. L. *et al.* Inhibitory cross-talk between the AMPK and ERK pathways mediates endoplasmic reticulum stress-induced insulin resistance in skeletal muscle. Br J Pharmacol 169, 69–81, 10.1111/bph.12124 (2013). 23373714 PMC3632239

[b88] Jager J. *et al.* Deficiency in the extracellular signal-regulated kinase 1 (ERK1) protects leptin-deficient mice from insulin resistance without affecting obesity. Diabetologia 54, 180–189, 10.1007/s00125-010-1944-0 (2011). 20953578

[b89] Miura S., Kai Y., Ono M. & Ezaki O. Overexpression of peroxisome proliferator-activated receptor gamma coactivator-1alpha down-regulates GLUT4 mRNA in skeletal muscles. J Biol Chem 278, 31385–31390, 10.1074/jbc.M304312200 (2003). 12777397

[b90] Michael L. F. *et al.* Restoration of insulin-sensitive glucose transporter (GLUT4) gene expression in muscle cells by the transcriptional coactivator PGC-1. Proc Natl Acad Sci USA 98, 3820–3825, 10.1073/pnas.061035098 (2001). 11274399 PMC31136

[b91] Handschin C. *et al.* Abnormal glucose homeostasis in skeletal muscle-specific PGC-1alpha knockout mice reveals skeletal muscle-pancreatic beta cell crosstalk. J Clin Invest 117, 3463–3474, 10.1172/JCI31785 (2007). 17932564 PMC2000810

[b92] Zechner C. *et al.* Total skeletal muscle PGC-1 deficiency uncouples mitochondrial derangements from fiber type determination and insulin sensitivity. Cell Metab 12, 633–642, 10.1016/j.cmet.2010.11.008 (2010). 21109195 PMC2999961

[b93] Misra P. *et al.* Phosphorylation of transcriptional coactivator peroxisome proliferator-activated receptor (PPAR)-binding protein (PBP). Stimulation of transcriptional regulation by mitogen-activated protein kinase. J Biol Chem 277, 48745–48754, 10.1074/jbc.M208829200 (2002). 12356758

[b94] Finck B. N. *et al.* A potential link between muscle peroxisome proliferator- activated receptor-alpha signaling and obesity-related diabetes. Cell Metab 1, 133–144, 10.1016/j.cmet.2005.01.006 (2005). 16054054

[b95] Jena P. K. *et al.* Impact of targeted specific antibiotic delivery for gut microbiota modulation on high-fructose-fed rats. Appl Biochem Biotechnol 172, 3810–3826, 10.1007/s12010-014-0772-y (2014). 24574250

