# Supplementary material for: Co-activator binding protein PIMT mediates TNF-α induced insulin resistance in skeletal muscle via the transcriptional down-regulation of MEF2A and GLUT4
Source: Sci Rep. 2015 Oct 15;5:15197. doi: 10.1038/srep15197 (PMC4606566; doi:10.1038/srep15197)

Co-activator binding protein PIMT mediates TNF-α induced insulin resistance in skeletal muscle via the transcriptional down-regulation of MEF2A and GLUT4

Vasundhara Kain[1£], Bandish Kapadia[1], Navin Viswakarma[1¤], Sriram Seshadri[2], Bhumika Prajapati[2], Prasant K Jena[2¥], Chandana Lakshmi Teja Meda[1], Maitreyi Subramanian[1], Sashidhara Kaimal Suraj[3], Sireesh T Kumar[3], Phanithi Prakash Babu[3], Bayar Thimmapaya[4], Janardan K Reddy[5], Kishore V. L. Parsa[1*], Parimal Misra[1]*

[1]Department of Biology, Dr. Reddy's Institute of Life Sciences, University of Hyderabad Campus, Hyderabad, Telangana, India, [2]Institute of Science, Nirma University, Sarkhej-Gandhinagar Highway, Ahmedabad, Gujarat, India, [3]Department of Biotechnology, School of Life Sciences, University of Hyderabad, Hyderabad, Telangana, India, [4]Department of Microbiology and Immunology, Feinberg School of Medicine, Northwestern University, Chicago, Illinois, United States of America, [5]Department of Pathology, Feinberg School of Medicine, Northwestern University, Chicago, Illinois, United States of America.

[£] Current address: Division of Cardiovascular disease, School of Medicine, The University of Alabama Birmingham, Alabama, USA.

¤ Current address: Department of Molecular Pharmacology and Therapeutics, Loyola University Chicago, Maywood, Illinois, USA

[¥]Current address: Department of Medical Pathology and Laboratory Medicine, University of California, at Davis Medical Center, Sacramento, California, United States of America.

* Correspondence to Kishore VL Parsa (kishorep@drils.org); Parimal Misra (parimalm@drils.org)

Supplemental Methods

Confocal Microscopy

L6 myoblasts were transiently transfected with pCMV-PIMT-Flag, pcDNA3.1-PIMT^{S298A} and pcDNA3.1-PIMT^{S298D} for 24 h, fixed with 3% paraformaldehyde, permeabilized with 0.02% Triton X-100 and blocked with 5% BSA, followed by treatment with primary rabbit polyclonal GLUT4 antibody (1:50) for 2 h at room temperature and Cy3-conjugated goat anti-rabbit antibody (1:500; Chemicon International, CA, USA) for 1 h. 100nM DAPI was used to stain the nuclei. Coverslips were mounted with antifade (Vector Laboratories, CA, USA) on glass slides and cellular images were acquired using a Leica SP2 AOBS Laser Confocal Microscope (Leica, IL, USA). Unstained cells served as negative control.

Supplemental figure legends:

Supplemental figure 1 | TNF-α abrogates basal and insulin stimulated glucose uptake. (a,b) Mean basal and insulin stimulated (5 min and 10 min) uptake of 2-NBDG by L6 myoblasts (a) and neonatal muscle cells (b) treated with TNFα for 48 h. Values are shown as the mean±SD after normalizing with the corresponding protein content and expressed relative to basal of control cells which was set to 1. **p*<0.05, ***p*<0.01, ****p*<0.005 versus control cells (two way ANOVA).

Supplemental figure 2 | PIMT mediates TNF-α induced insulin resistance in skeletal muscle cells. **(a)** Mean basal and insulin stimulated (5 min and 10 min) uptake of 2-NBDG by L6 myoblasts. Values are shown as mean±SD after normalizing with the corresponding protein content and expressed relative to basal of control cells which was set to 1; ****p*<0.005 versus corresponding control cells (two way ANOVA). (b) mRNA expression of rattus PIMT in L6 myoblasts transfected with control siRNA (control si) or two different PIMT siRNA (PIMT si1 and PIMT si2). Values are shown as mean±SD; ****p*<0.005. (c) Western blotting to detect levels of IRS^{Ser307}, IRS^{Tyr608}, Total IRS, pAkt, phospho-p38, total p38 and β-actin in adeno injected (LacZ or PIMT) L6 myoblasts cultured with BSA or TNF-α (48 h), treated with or without insulin (100nM) for 30 min. The cropped blots were run under the same experimental conditions. The full-length blots are included in Supplemental Figure 8.

Supplemental figure 3 | Enhanced PIMT expression reduces glucose uptake in neonatal myoblasts . Mean basal and insulin stimulated (5 min and 10 min) uptake of 2-NBDG into neonatal muscle cells infected with Ad-PIMT or Ad-$PIMT^{S289A}$. Ad-EGFP infected cells and un-infected cells were used as internal control. Values are shown as the mean±SD after normalizing with the corresponding protein content and expressed relative to basal of un-infected control cells which was set to 1. **p*<0.05, ***p*<0.01 versus control cells, $^{@}p$<0.05, $^{\$}p$<0.01 versus Ad-PIMT infected cells (two way ANOVA).

Supplemental figure 4 | qPCR analysis to confirm overexpression of human PIMT in L6 myotubes. mRNA expression of human PIMT (hPIMT) in Ad-PIMT, Ad-PIMT ser^{298} mutant infected L6 myotubes. Ad-Lacz served as the control.

Supplemental figure 5 | Overexpression of PIMT reduces GLUT4 levels. (a) mRNA expression of GLUT4 in L6 myoblasts transfected with PIMT(WT and Ser^{298} mutants). Values are shown as mean±SD; ****p*<0.005 versus control cells, $^{\$}p$<0.005 versus PIMT overexpressing cells. (b) Confocal microscopy for detection and localization of GLUT4 (anti-GLUT4-Cy3) in un-treated PIMT (WT and Ser^{298} mutants) overexpressing L6 myoblasts. Nuclei were stained by DAPI (blue).

Supplemental figure 6 | Ectopic expression of PIMT modulates the expression of MEF2 family members in Ser^{298} phosphorylation dependent manner. (a-d) mRNA expression of rattus MEF2A (a), MEF2D (b), MEF2C (c) and PGC-1α (d) in L6 myoblasts transfected with PIMT WT or PIMT Ser^{298} mutants. Values are shown as the mean±SD; *****p*<0.001 versus control cells, $^{\$}p$<0.005 versus PIMT (WT) transfected cells (two way ANOVA).

Supplemental figure 7 | Validation of overexpression of hPIMT in the skeletal muscle of Wistar rats. (a) Fluorescence microscopic image of rat skeletal muscle tissue to confirm overexpression of PIMT EGFP. (b) mRNA expression of human PIMT in Ad-PIMT EGFP and Ad-PIMT Ser^{298} mutants infected rat skeletal muscle tissue. Values are shown as mean±SD.

Supplemental figure 8: Full images of the Western blot and gel pics

Supplemental Figure 1

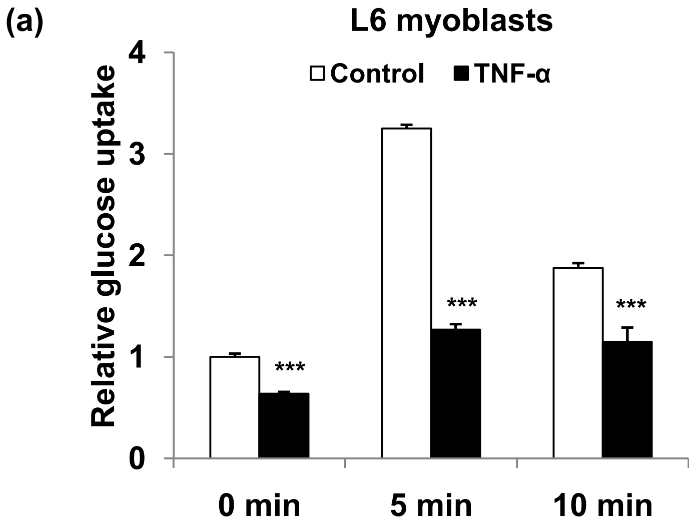

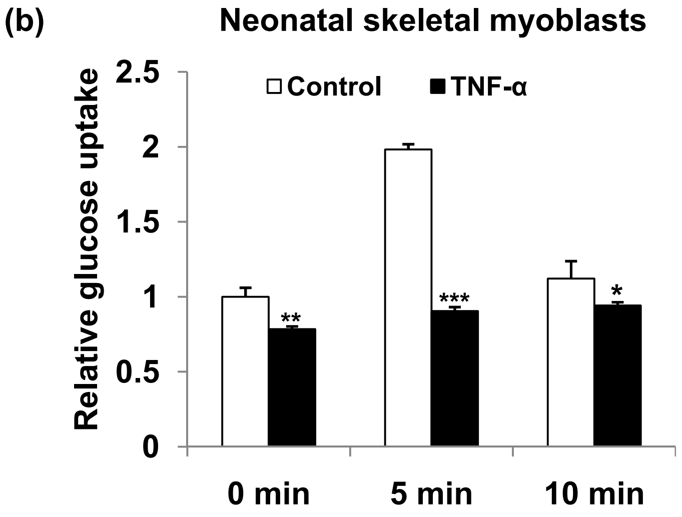

Supplemental Figure 2

(a)

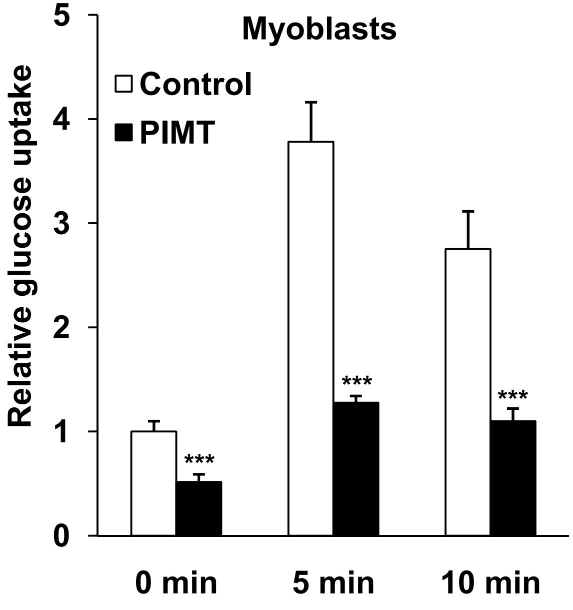

(b)

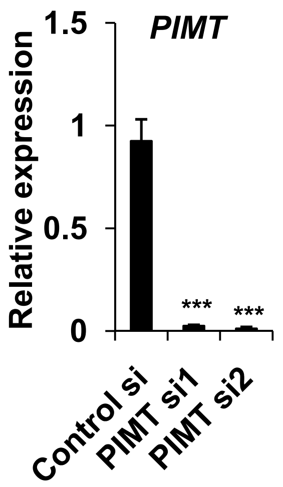

(c)

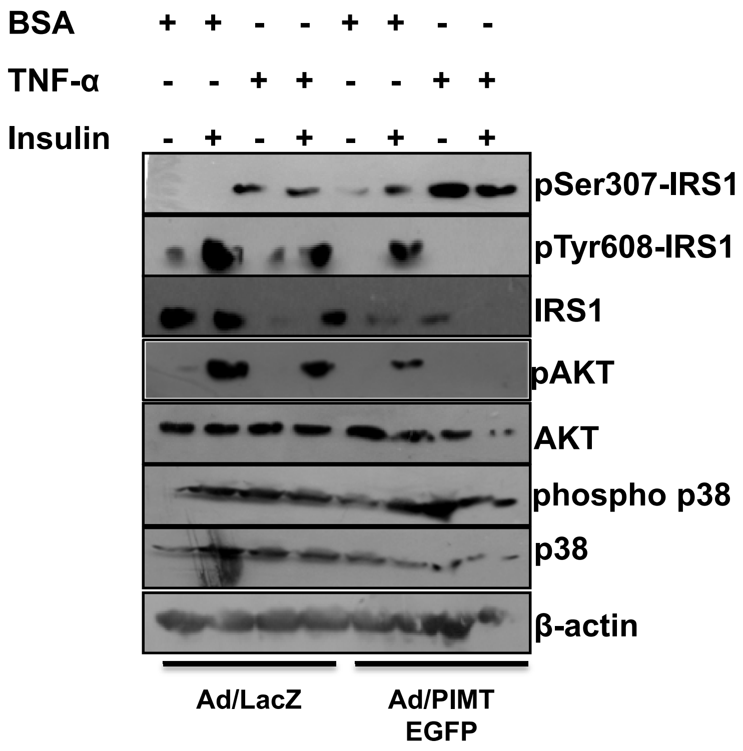

Supplemental Figure 3

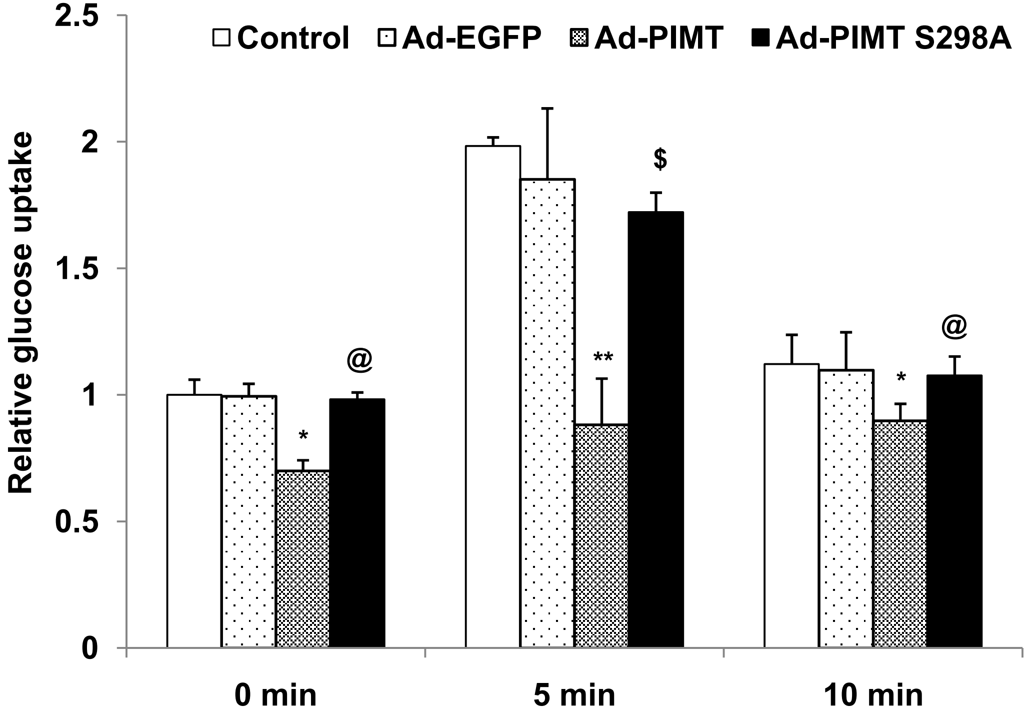

Supplemental Figure 4

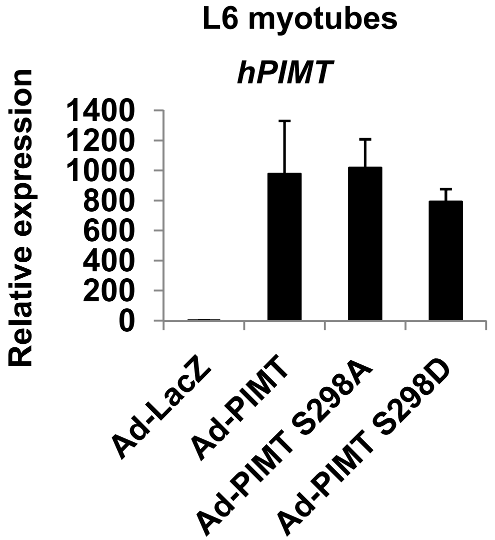

Supplemental Figure 5

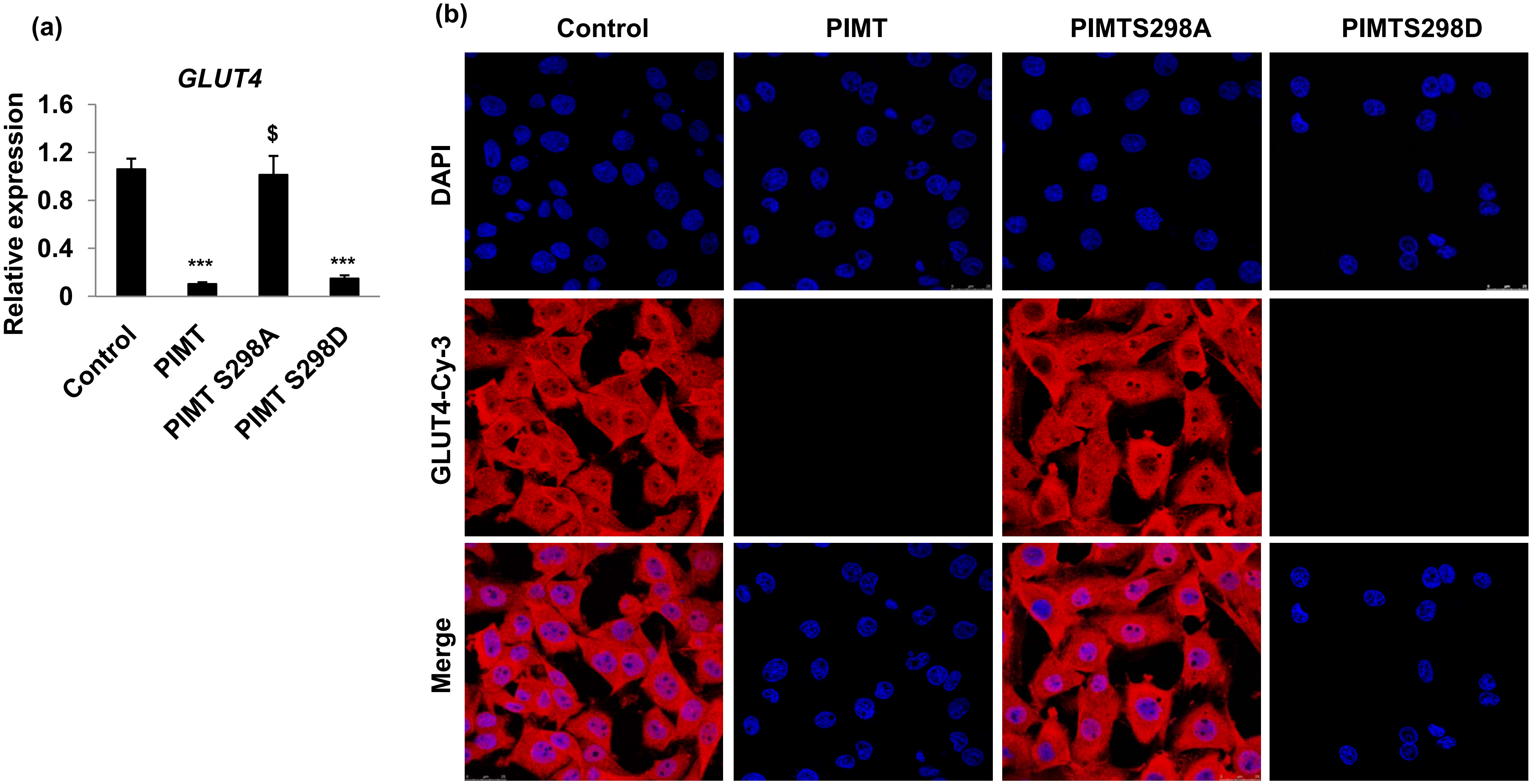

Supplemental Figure 6

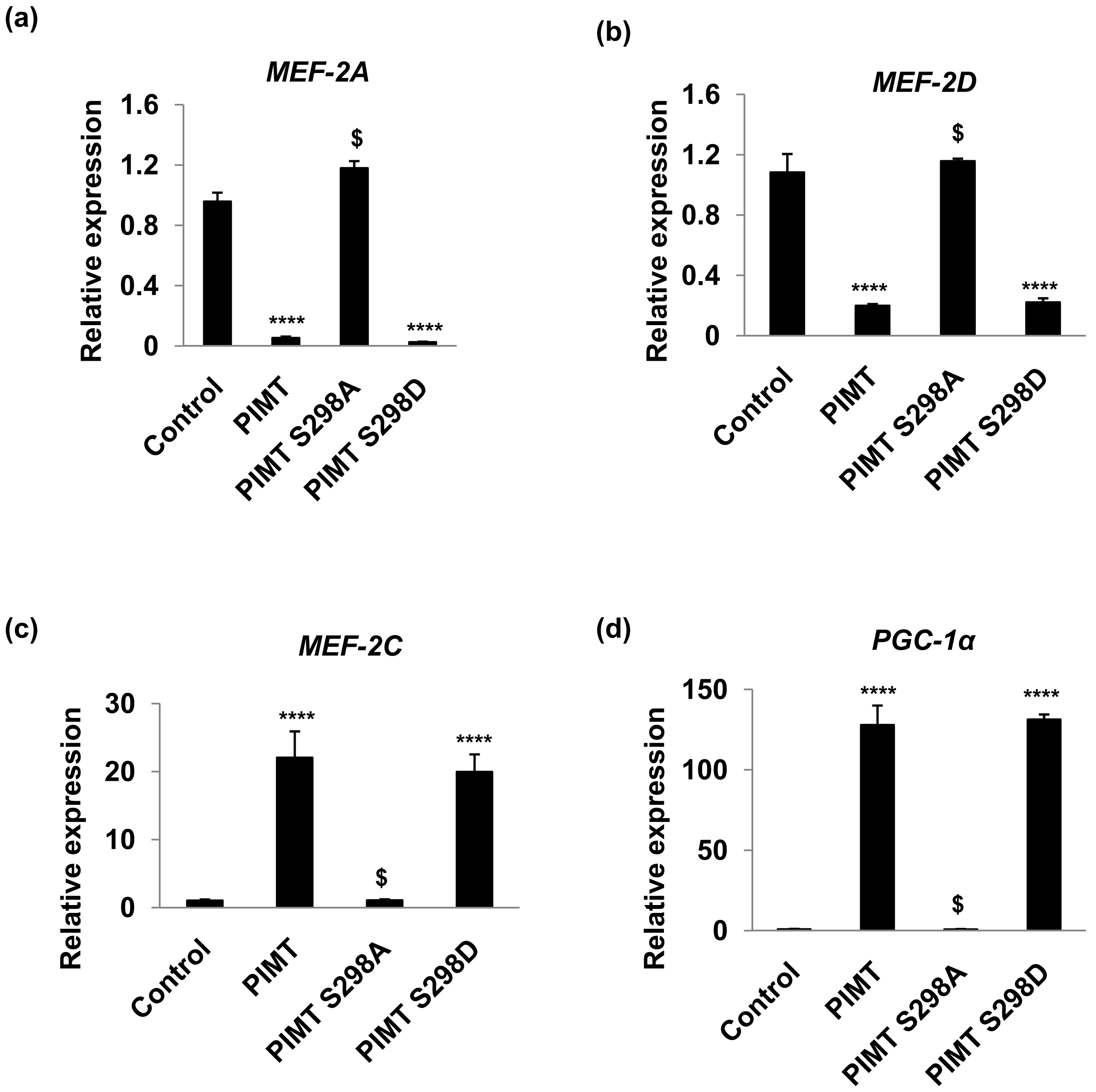

Supplemental Figure 7

(a)

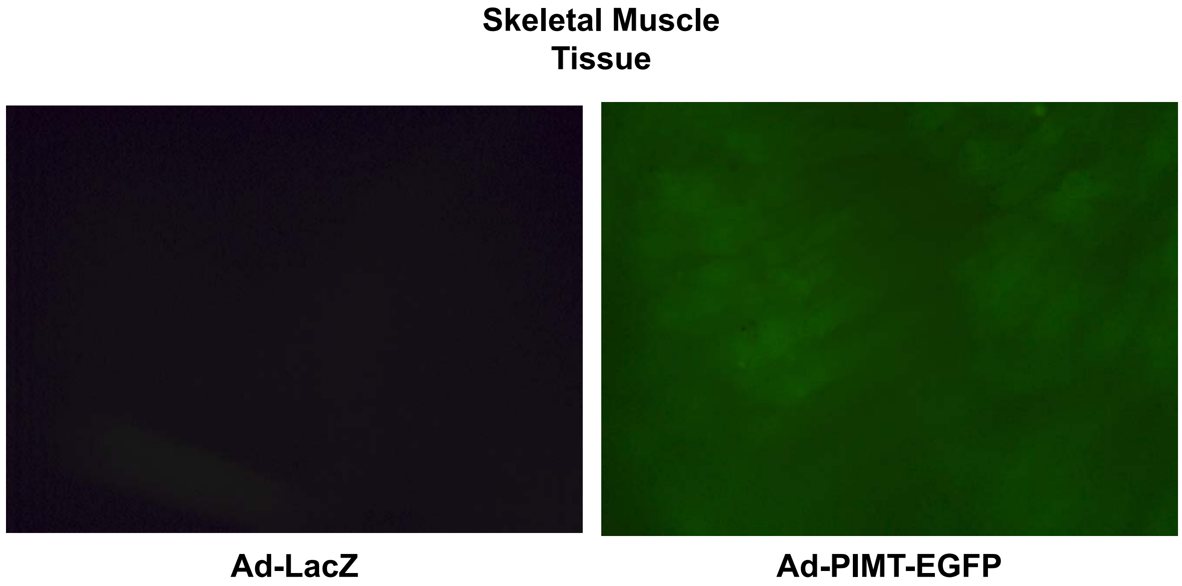

(b)

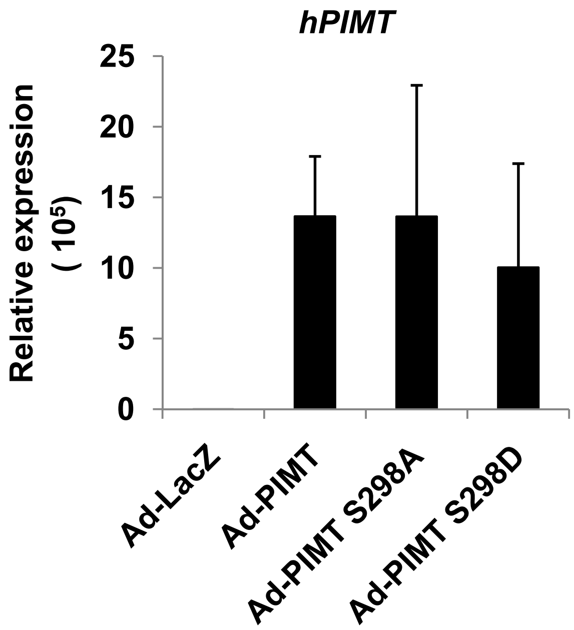

Supplemental Figure 8

Figure 1J

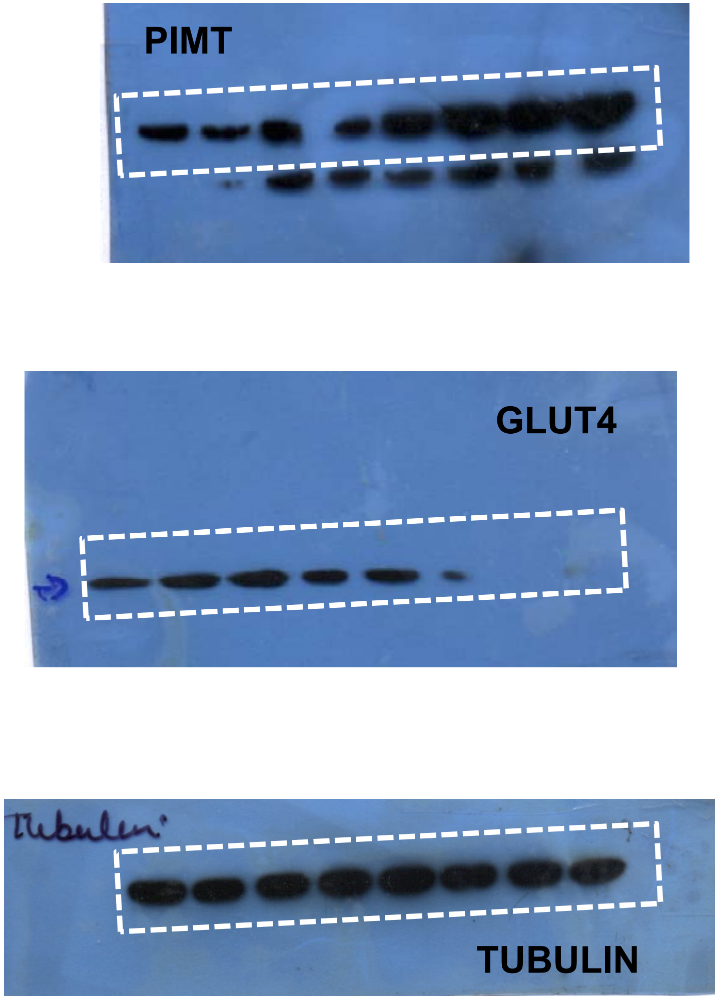

Figure 1H

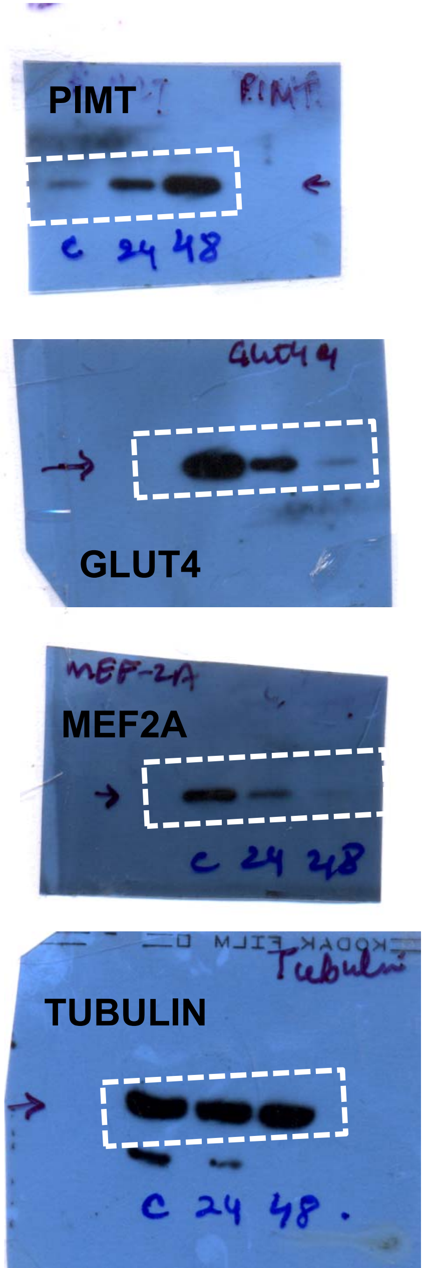

Supplemental Figure 8

Figure 2e

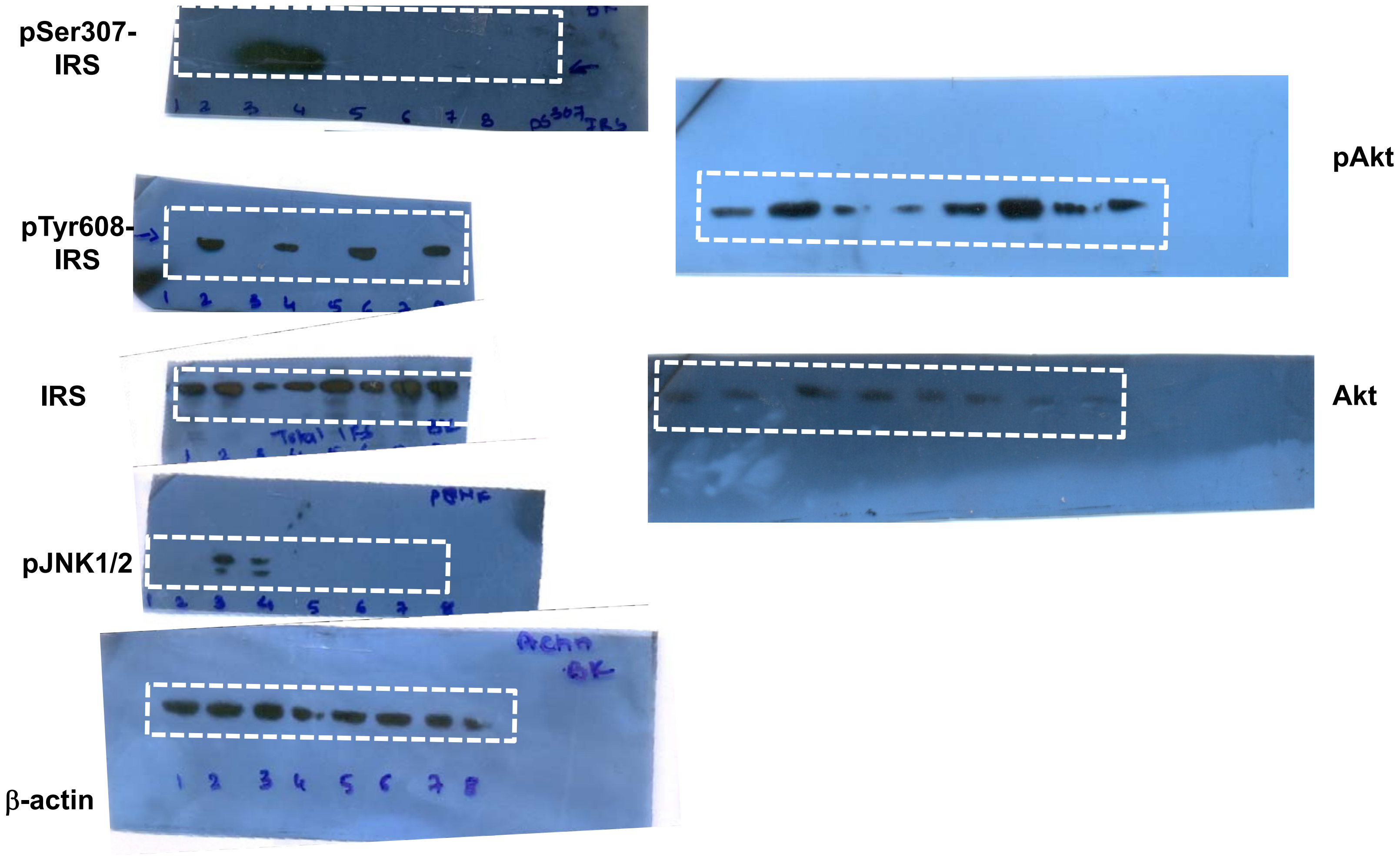

Supplemental Figure 8

Figure 3A

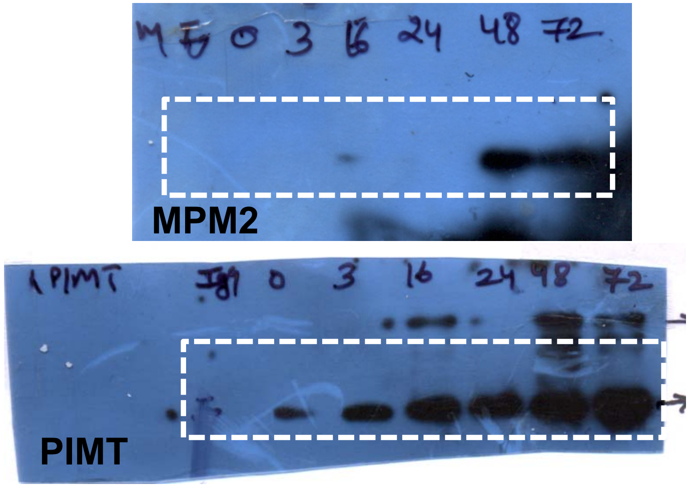

Figure 4C

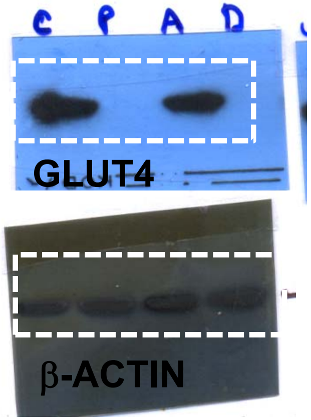

Figure 4E

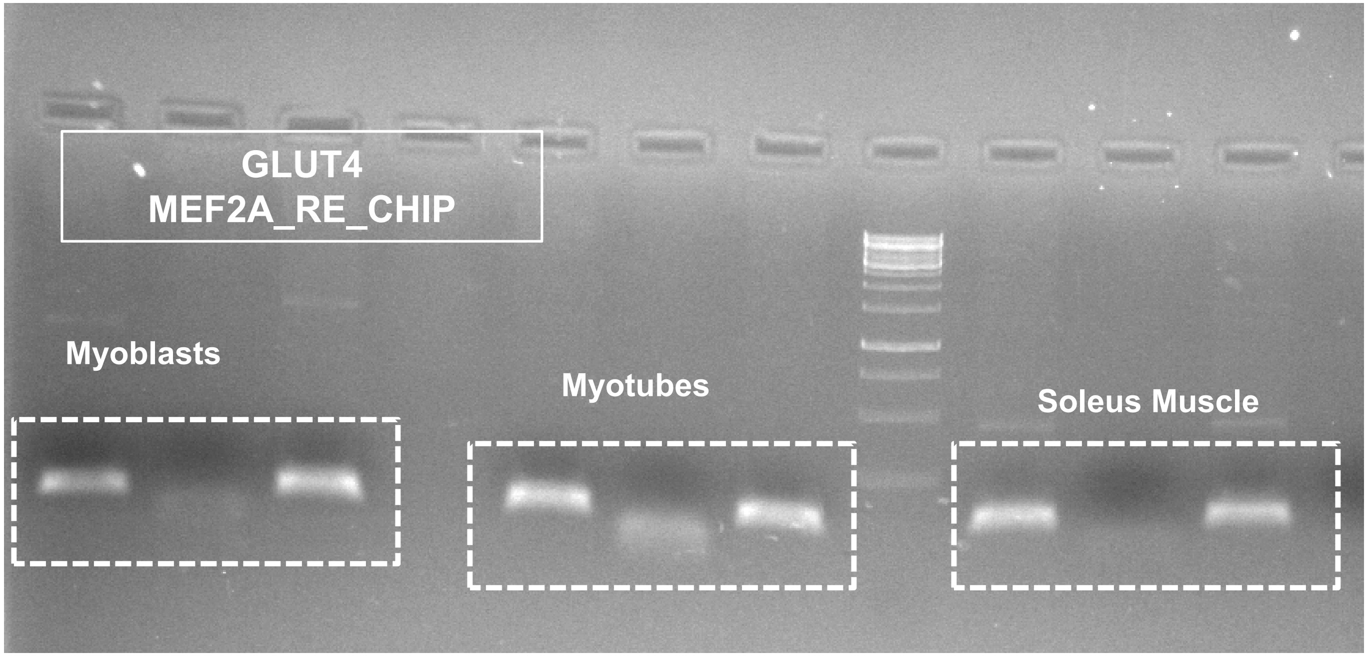

Supplemental Figure 8

Figure 5J

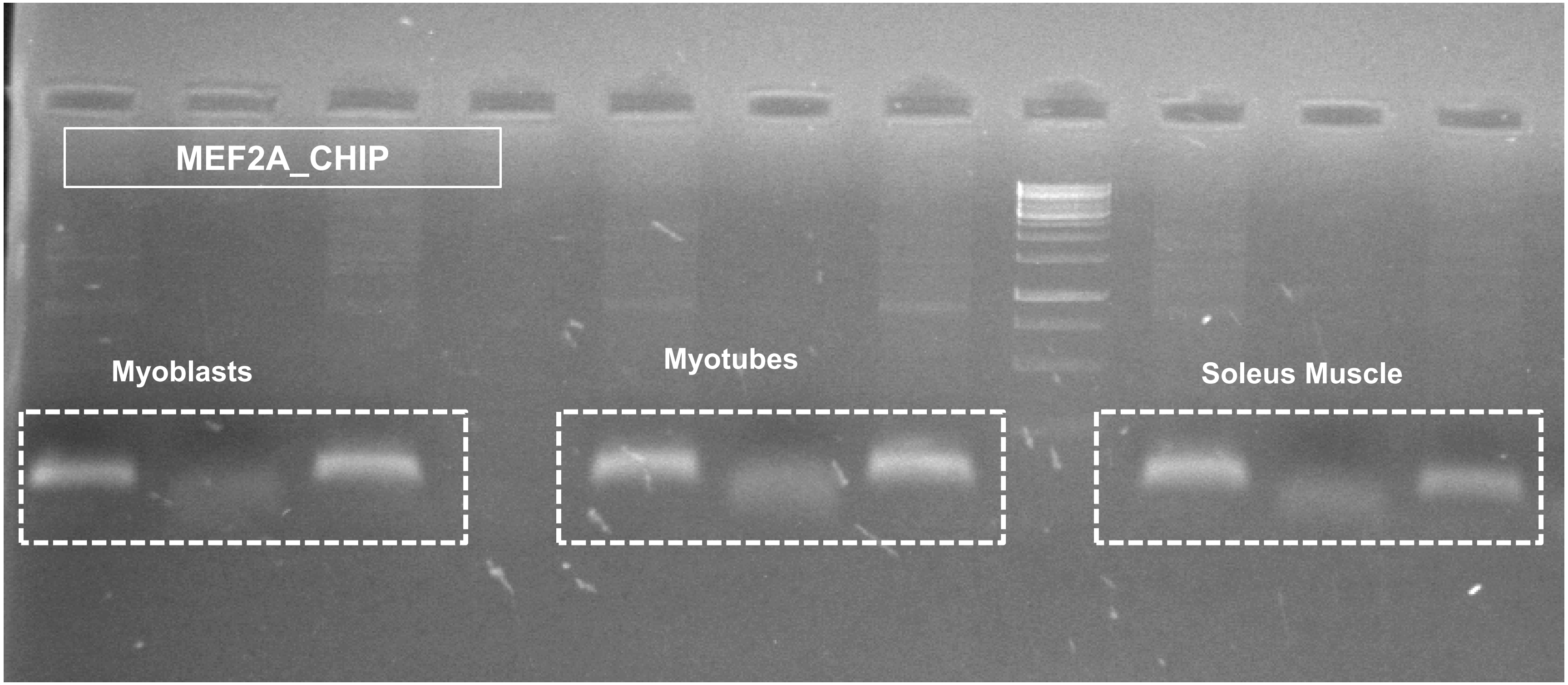

Supplemental Figure 8

Figure 6E

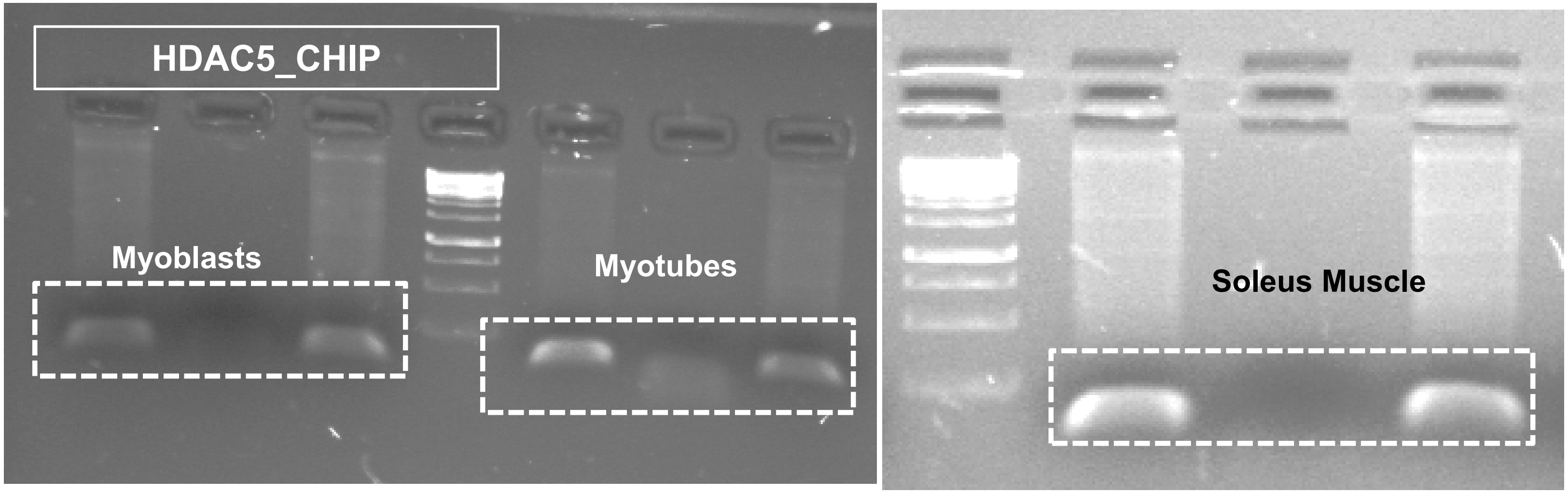

Figure 6F

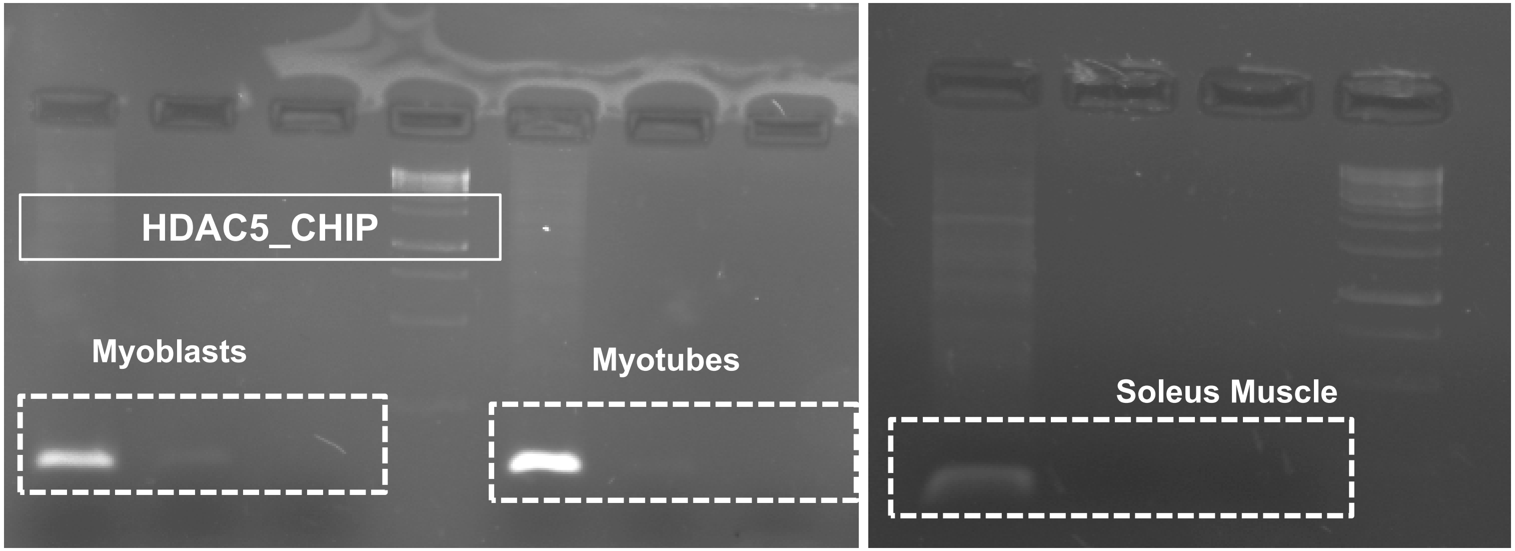

Figure 7A

Supplemental Figure 8

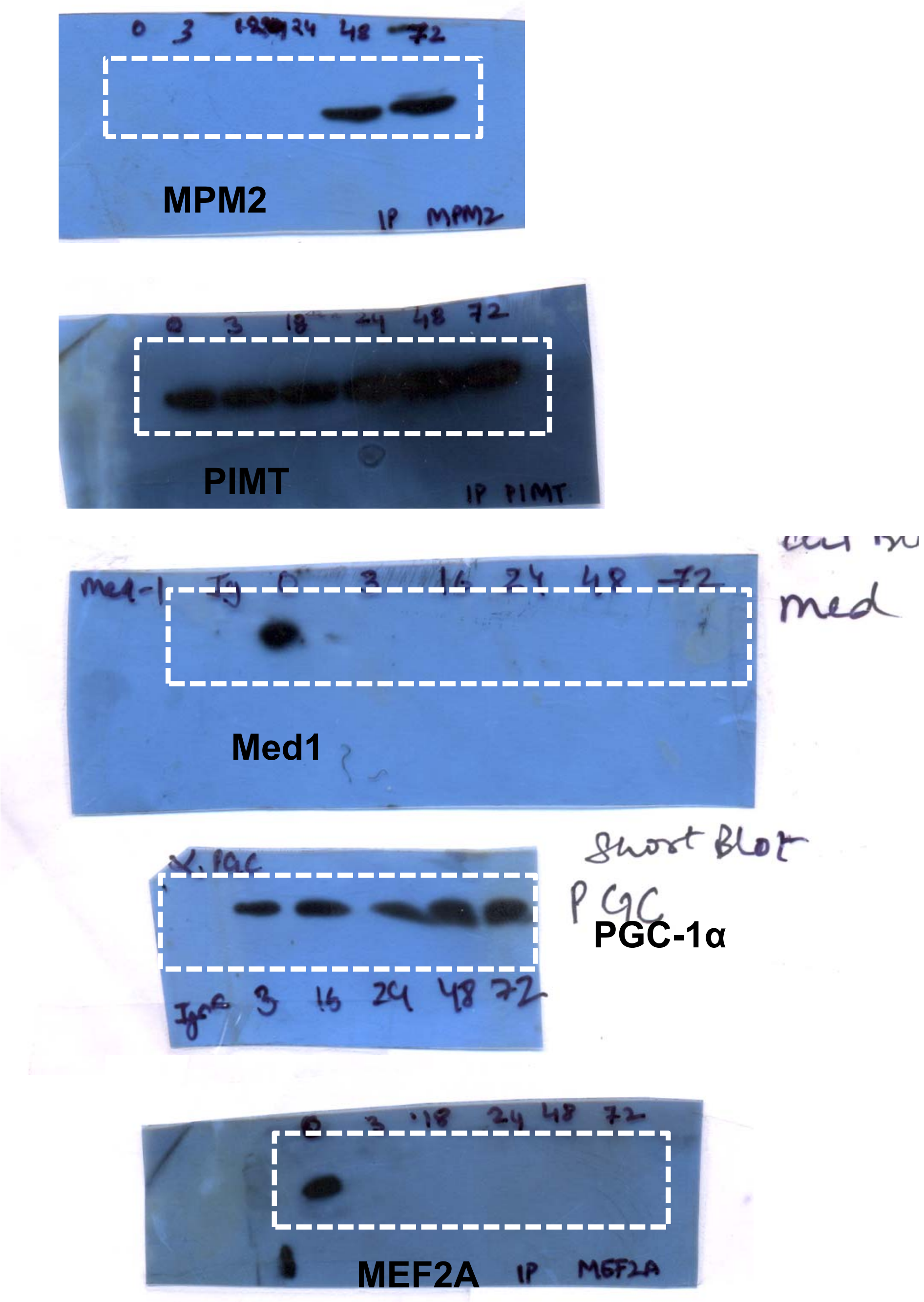

Figure 7C

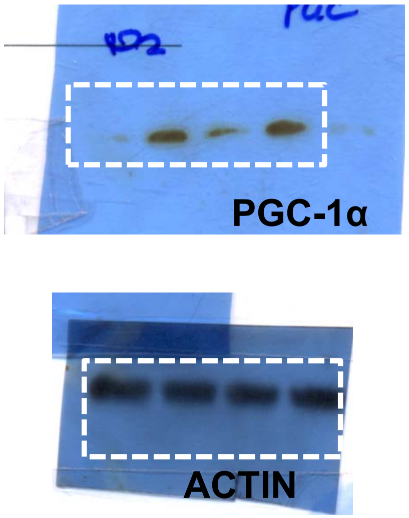

Supplemental Figure 8

Supplemental Figure 2c

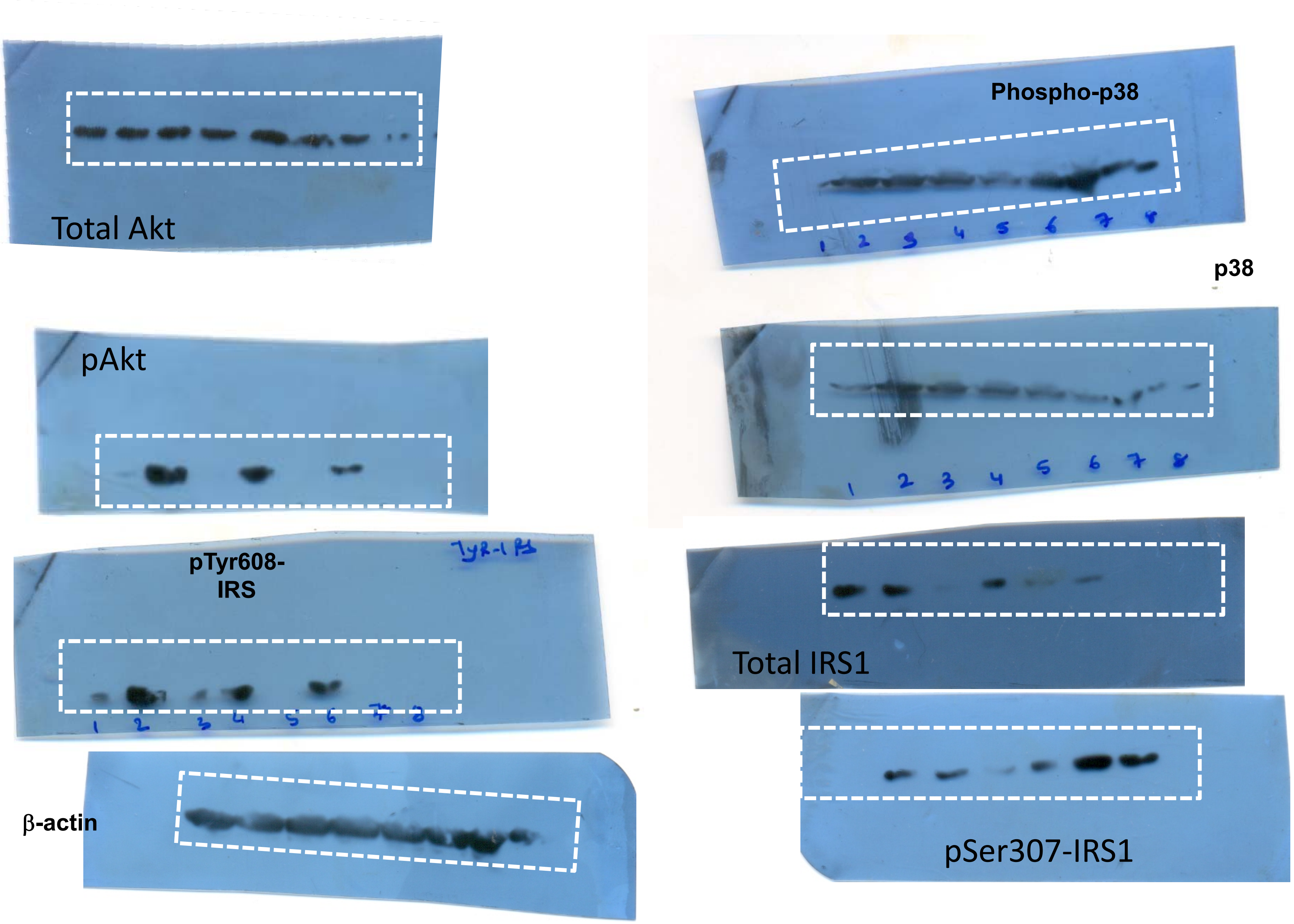

Supplement: Supplementary Information [file srep15197-s1.pdf]
